# Supine Bridge Exercise: A Narrative Review of the Literature (Part I)

**DOI:** 10.7759/cureus.80349

**Published:** 2025-03-10

**Authors:** Saverio Colonna, Antonio D'Alessandro, Riccardo Tarozzi, Fabio Casacci

**Affiliations:** 1 Rehabilitation Medicine, Spine Center, Bologna, ITA; 2 Research and Development, Osteopathic Spine Center Education (OSCE), Bologna, ITA; 3 Education, Osteopathic Spine Center Education (OSCE), Bologna, ITA

**Keywords:** barbell glute bridge, barbell hip thrust, erector spinae, external oblique muscle, gluteus maximus muscle, gluteus medius, hamstring muscle, multifidus, supine bridge exercise

## Abstract

This article represents the first part of a larger work aimed at exploring the use of the supine bridge exercise (SBE) for both therapeutic and preventive purposes concerning lower back and hip joint pathologies, which will be presented in a subsequent article.

The current article presents various execution modes of SBE found in the literature. It discusses what is involved in performing the traditional SBE with different angles at the ankle, knee, hip, and spine. It also addresses SBE variations, such as (1) single-leg; (2) with simultaneous activation of the hip adductors; (3) with simultaneous activation of the hip abductors; (4) with elevated upper trunk support; and (5) with foot-elevated support on a stable or unstable surface. Additionally, it reviews the literature on how different methods of performing the SBE engage muscles responsible for the stability of the hip, pelvis, and lumbar spine, including the gluteus maximus, gluteus medius, hamstrings, erector spinae, multifidus, transversus abdominis, and external oblique.

The aim of this article is to serve as a practical guide or "manual" for utilizing SBE across a variety of rehabilitative contexts, providing insights into how the exercise can be adapted to target specific muscles effectively in different clinical scenarios.

## Introduction and background

In recent years, the importance of core stability and strength has been widely recognized in rehabilitation and athletic training [1]. The core muscles act as the center of the functional kinetic chain, contribute to resisting spinal disturbances [2], and transfer power to the distal segments during athletic activities [3]. In rehabilitation, core strengthening exercises can reduce the risk of injury by increasing muscle power and endurance [4]. Typical core strengthening exercises include the supine bridge, prone bridge, and crunch exercises [5].

The bridge exercise is one of the most studied and used exercises in rehabilitation. For instance, searching for "bridge exercise" AND "rehabilitation" or "bridge exercise" AND rehabilitation AND "muscle activation" on Google Scholar returns approximately 1500 articles; meanwhile, the PubMed search ("bridge exercise" AND rehabilitation; "bridge exercise" AND rehabilitation AND effectiveness) for the bridge exercise returns 74 articles. Given the vast amount of literature, this review focuses solely on the supine bridge exercise (SBE). In the literature, this exercise is recommended for preventing injuries to the hamstrings [6], iliopsoas tendinitis [7], and chronic nonspecific low back pain (LBP) [8]. Additionally, there is extensive literature reporting various execution methods and the major muscle groups involved.

This article aims, starting from biomechanics, to delve into the execution methods of SBE proposed in the literature through a narrative review. This review serves as the foundation for a second article (Part II), which will present how SBE is applied to treat certain pathologies. Furthermore, starting from physiology, a new model for applying SBE to issues of the lumbar spine and hip joint will be introduced.

The SBE is a multi-joint, closed-kinetic-chain, bodyweight exercise that can also be performed on unstable surfaces [9] and with additional external resistance [10,11]. From a kinematic perspective, the classic bridge exercise has one degree of freedom. The body configuration in each phase of the exercise, considering the starting position and the subject's anthropometry, is entirely determined by one parameter, usually represented by the elevation of the pelvis or the inclination of the torso relative to the horizontal [12]. There are many ways to perform the supine bridge, as reported in the literature. Given the difficulty in addressing them all, we will focus on just a few that we believe are most relevant to hip and lumbar spine pathologies. While the SBE has a relatively standardized setup, its variations can significantly alter the load on the musculoskeletal structures involved, making them more or less suitable or contraindicated for the treatment or prevention of certain musculoskeletal pathologies.

Analyzing what is reported in the literature through a narrative review, we will start by discussing the classic execution of the SBE, then move on to some variations that may involve the joint angles of the ankle, knee/hip, and lumbar spine and the use of aids such as benches, balls, or elastic bands to elevate the support points and/or create instability.

And finally, we discuss a review of the different methods of performing the SBE, as proposed in the literature, and the related activation of the gluteus maximus (GM), gluteus medius (Gmed), hamstrings (Ham), erector spinae (ES), multifidus (MF), transversus abdominis (TrA), and external oblique (EO).

## Review

Execution of the traditional SBE

**Figure 1:**
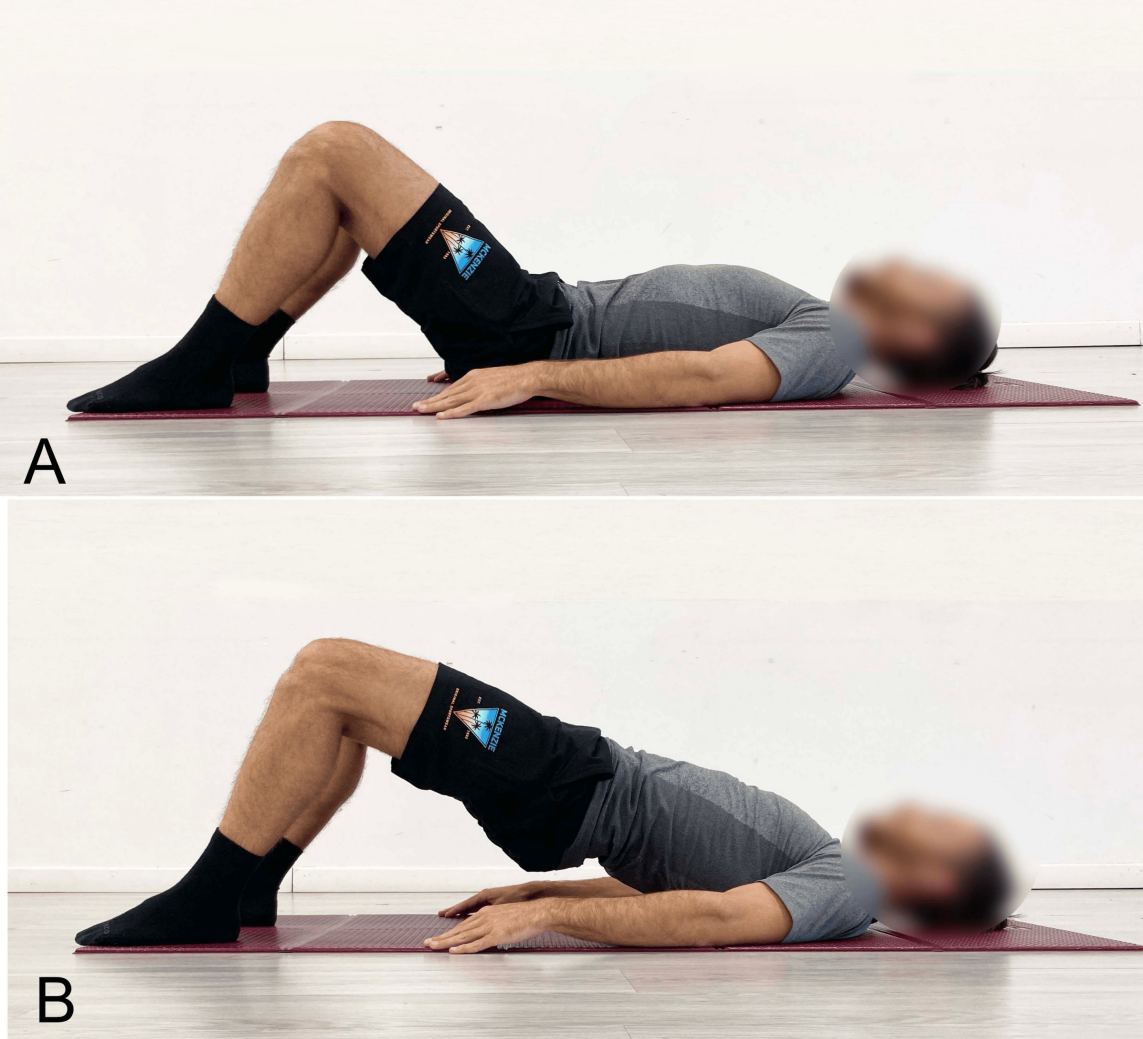
Example of performing the classic supine bridge exercise A) Starting position; B) End position. Image credit: Author Saverio Colonna

In the starting position of the classic SBE, the subject lies supine with the hips and knees bent and the feet placed on the ground at hip-width apart (Figure 1A). The individual depicted in all images included in this publication provided written informed consent for the use of his images.

During the concentric phase of the exercise, while maintaining the upper spine in contact with the ground, the pelvis is lifted from the floor until it reaches the neutral angular position of the hip, using the push of the feet against the ground (Figure 1B). Once this position is achieved, it can be held isometrically for a set time, or the eccentric phase can immediately follow, during which the pelvis is lowered back to the floor.

Possible variations of the traditional SBE

The following section will discuss how positional changes of the involved segments during the execution of the traditional SBE lead to varying myofascial involvement.

Despite the popularity of the supine bridge, only one study [12] had, to our knowledge, provided a quantitative biomechanical analysis of this exercise. The SBE is characterized by a closed kinetic chain at both ends, represented by the support of the cervical-thoracic spine and the feet, consisting of three segments: the leg, thigh, and trunk. During execution, it is recommended [12] to globally stabilize the spine in the neutral position, so the trunk is considered as a single segment. This type of chain has a single degree of freedom, and as a closed kinetic chain, the movement occurring at one joint is transferred to the other joints involved [12]. The distance between the feet and the gluteal contact with the floor influences the initial flexion of the joints involved, constituting the primary variable of the traditional exercise. The functions linking the angles of the ankle, knee, and hip are not well understood, and understanding these functions is essential for calculating the torque developed by the muscles at different joints and during different phases of the exercise. For the knee, for example, depending on the working angle, the torque shifts from the posterior thigh muscles (Ham) to the anterior muscles (quadriceps), while at the hip, an extensor torque is always present [12].

**Figure 2:**
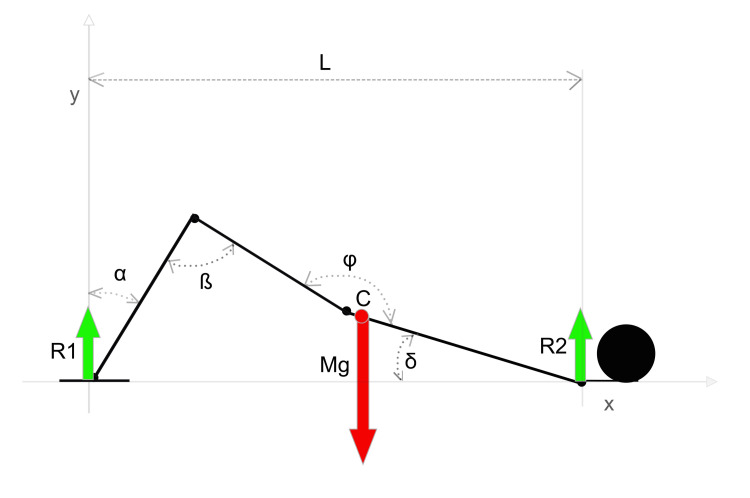
Biomechanical diagram of the supine bridge exercise Mg: body weight vector applied at the center of body mass (C); R1: ground reaction vector at the foot level; R2: ground reaction vector at the dorsal contact point; α: angle at the tibio-tarsal level; β: angle at the knee level; φ: angle at the hip level; δ: angle between the trunk axis and the ground (trunk tilt); L: distance between the foot contact point and the dorsal contact point (thoraco-podalic distance). Adapted from Biscarini et al. [12]. Image credit: Author Saverio Colonna

As proposed by Biscarini et al. [12], the external forces acting on the body performing the exercise include its body weight and the ground reaction forces acting at the contact points between the body and the ground. The body weight is equivalent to a vector Mg applied at the center of mass of the body. The ground reaction forces can be represented as R1 at the foot level and R2 at the contact point of the upper trunk with the floor. The equilibrium equation of forces applied to the entire body, represented on Cartesian axes, is R1 + R2 - Mg = 0 (Figure 2).

Ankle Variable

**Figure 3:**
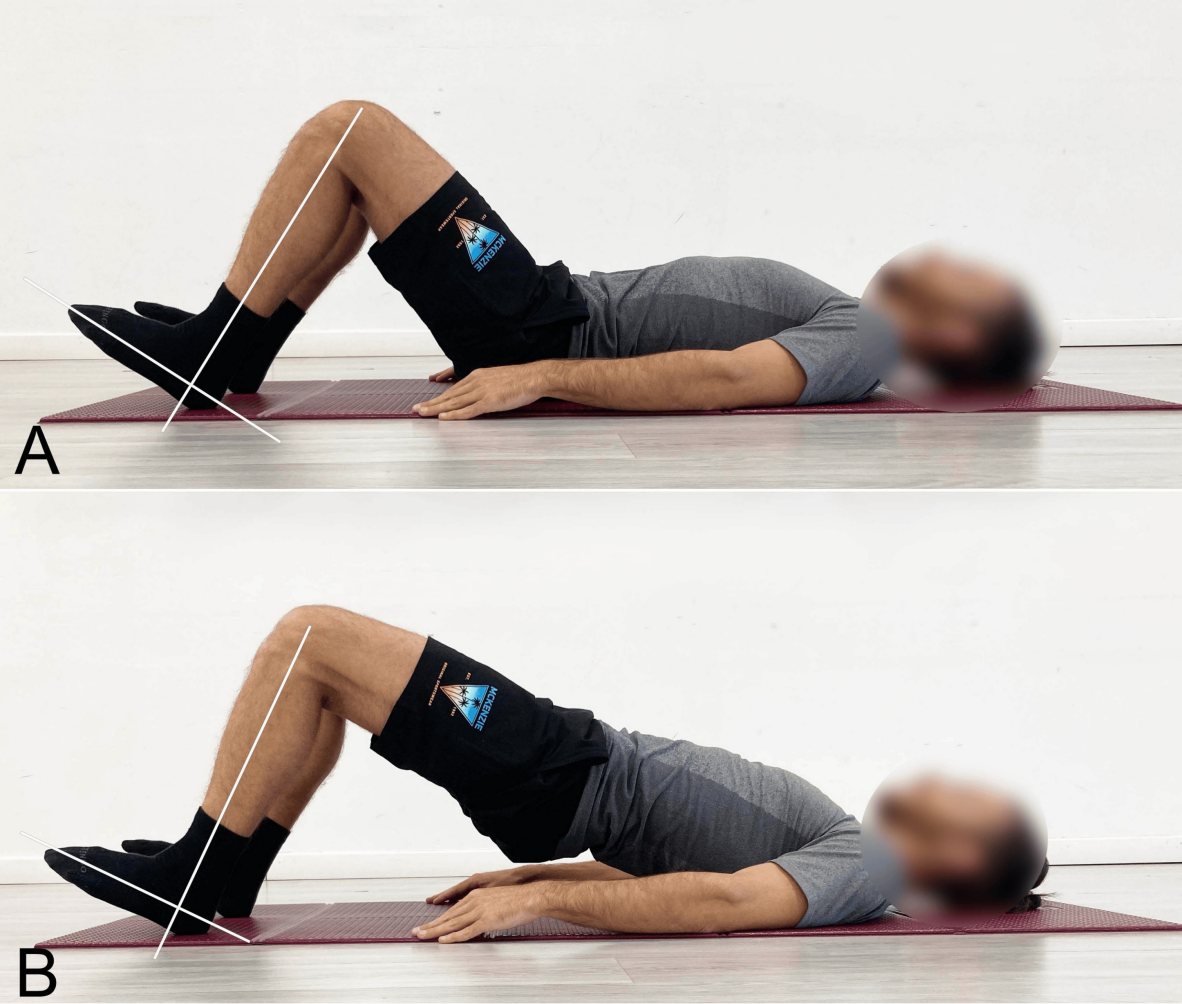
Ankle dorsiflexion variation of the supine bridge exercise A) Starting position; B) End position. Image credit: Author Saverio Colonna

Verstegen and Williams [13] suggest, without conducting specific research, that performing these bridging exercises with the forefoot elevated and only the heels in contact with the ground (ankle dorsiflexion, or DF) (Figures 3A, 3B) recruits the GM to a greater extent than bridging with the entire foot flat on the ground (ankle plantarflexion, or PF). They also suggest that bridging in PF recruits the Ham muscles to a greater extent than bridging in DF.

The only studies known to us that have examined lower limb muscle activity during bridging with different foot positions were conducted by Yoo [14], Escamilla et al. [15], and Lehecka et al. [16]. These studies analyzed the electromyographic activity (EMG) of the Ham and GM during traditional bridging, comparing the response when performed with the ankle in DF and PF. For this type of the surface, EMG used in research and present in the literature, the absolute EMG detected during the exercise from the tested muscles is not considered; instead, the comparison is expressed as a percentage relative to the maximum voluntary isometric contraction (MVIC) performed during another standardized exercise specifically targeting the evaluated muscle group.

According to Yoo [14], when bridging is performed by lifting the heels off the ground, compared to bridging with the feet fully flat on the ground, Ham activity is significantly lower, while GM activity is significantly higher.

Escamilla et al. [15] compared the execution of single- and double-leg bridging with the feet flat on the ground versus lifting the forefoot. The authors concluded that when the goal is to maximize Ham recruitment, PF (feet flat) is more effective than DF (forefoot lifted). Conversely, when the goal is to maximize recruitment of the quadriceps, hip adductors, and abdominal oblique muscles, DF is more effective than PF.

Lehecka et al. [16] reported significant changes in the activation of hip muscles, as measured electromyographically, with different ankle positions. Ankle DF, in a position where the knee is flexed at 90°, significantly reduced the activity of the biceps femoris (BF), decreasing from 69.18% of MVIC in the feet-flat position to 58.71% MVIC when the ankle was dorsiflexed, with the forefoot lifted off the ground.

Knee Variable

At the knee level, to maintain the position of the classic bridge, a knee flexion torque is required at the lowest trunk tilt (TT) values relative to the ground and at the highest values of the distance between the two contact points on the ground (thoraco-podalic distance, or TPd, represented as L in Figure 2). This distance is measured from the feet to the dorsal support point, typically occurring in the interscapular area, approximately at the T3 level [12]. Conversely, a knee extension torque is required at higher TT values and lower TPd values.

Specifically, during the standard bridge exercise, a knee flexion torque is always required at the start of the concentric phase of the exercise, when the pelvis is still close to the ground, regardless of the TPd value. According to Biscarini et al. [12], as the pelvis is progressively lifted off the ground, the knee flexion torque decreases, transitioning to a knee extension torque in the group of subjects studied for TPd < 1.0 m. Only when the feet are positioned farther from the pelvis (TPd ≥ 1.0 m) is a knee flexion torque maintained throughout the entire movement [12].

Using 15 healthy subjects, Takeshita et al. [17], using musculoskeletal model simulations, evaluated changes in the activation of the GM, ES, Ham, and adductor magnus as the knee angle was modified during the SBE. The authors conclude that knee flexion positioning during the bridging exercise has varying effects on joint and muscular forces around the hip joint and lumbar spine: as the knee joint angle increases, activation of the GM and adductor magnus tends to increase, while BF activation significantly decreases.

**Figure 4:**
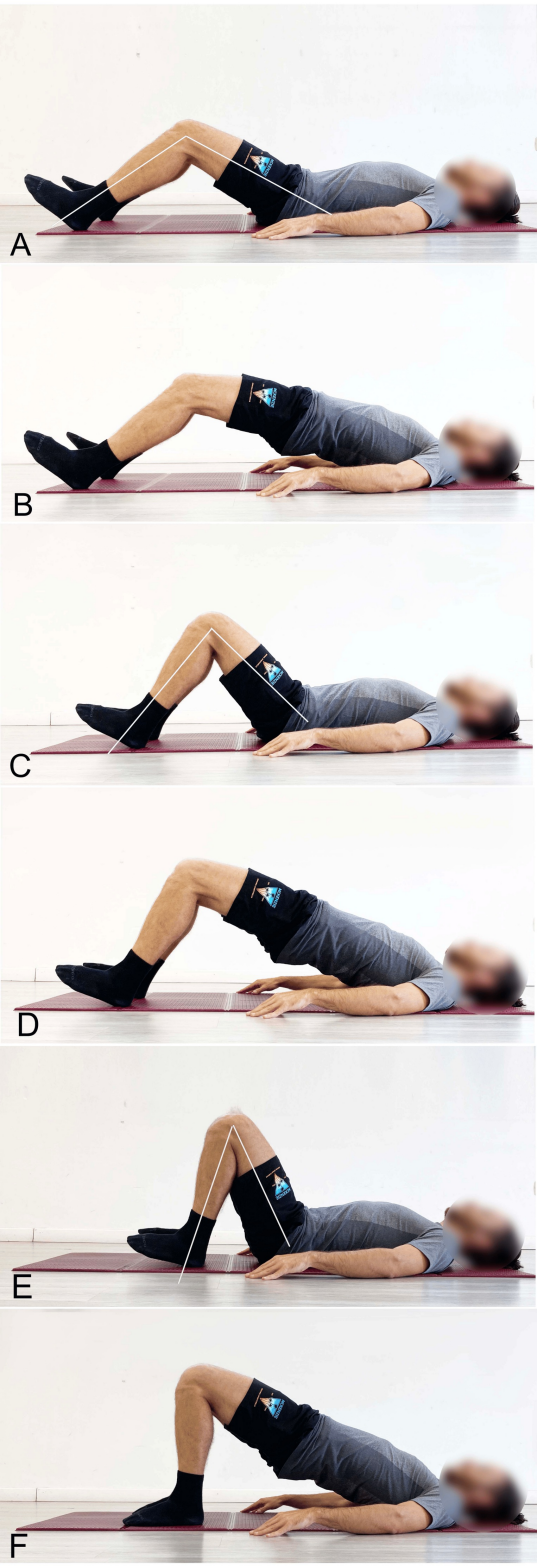
Example of supine bridge exercise with different knee flexion angles A) 60° starting position; B) 60° end position; C) 90° starting position; D) 90° end position; E) 120° starting position; F) 120° end position. Image credit: Author Saverio Colonna

Other studies confirm that during the SBE, as knee flexion increases (Figures 4A-4F), achieved by bringing the feet closer to the glutes, gluteal muscle activation increases, and Ham activation decreases [12,18].

Researchers [19] report GM activation during the SBE with bipodal support on a stable surface and a neutral spine of approximately 27% MVIC when the exercise is performed with flexed knees. In contrast, under the same neutral spine conditions but with extended knees and both feet on a Swiss ball, GM activation drops to 20% MVIC.

Several studies have assessed gluteal activation during the classic single-leg SBE, reporting fairly consistent results: Boren et al. [20] observed 54% MVIC for the GM and 54% MVIC for the Gmed; Ekstrom et al. [5] reported 40% MVIC for the GM and 47% MVIC for the Gmed; and Lehecka et al. [16] found 51.01% MVIC for the GM and 57.81% MVIC for the Gmed.

The modified single-leg bridge position, with 135° of knee flexion - potentially referred to as the "glute bridge" - demonstrated preferential activation of the GM and Gmed compared to the BF [16]; BF activity decreases from 75.34% MVIC in the traditional position to 23.49% MVIC in the hyper-flexed knee position.

**Figure 5:**
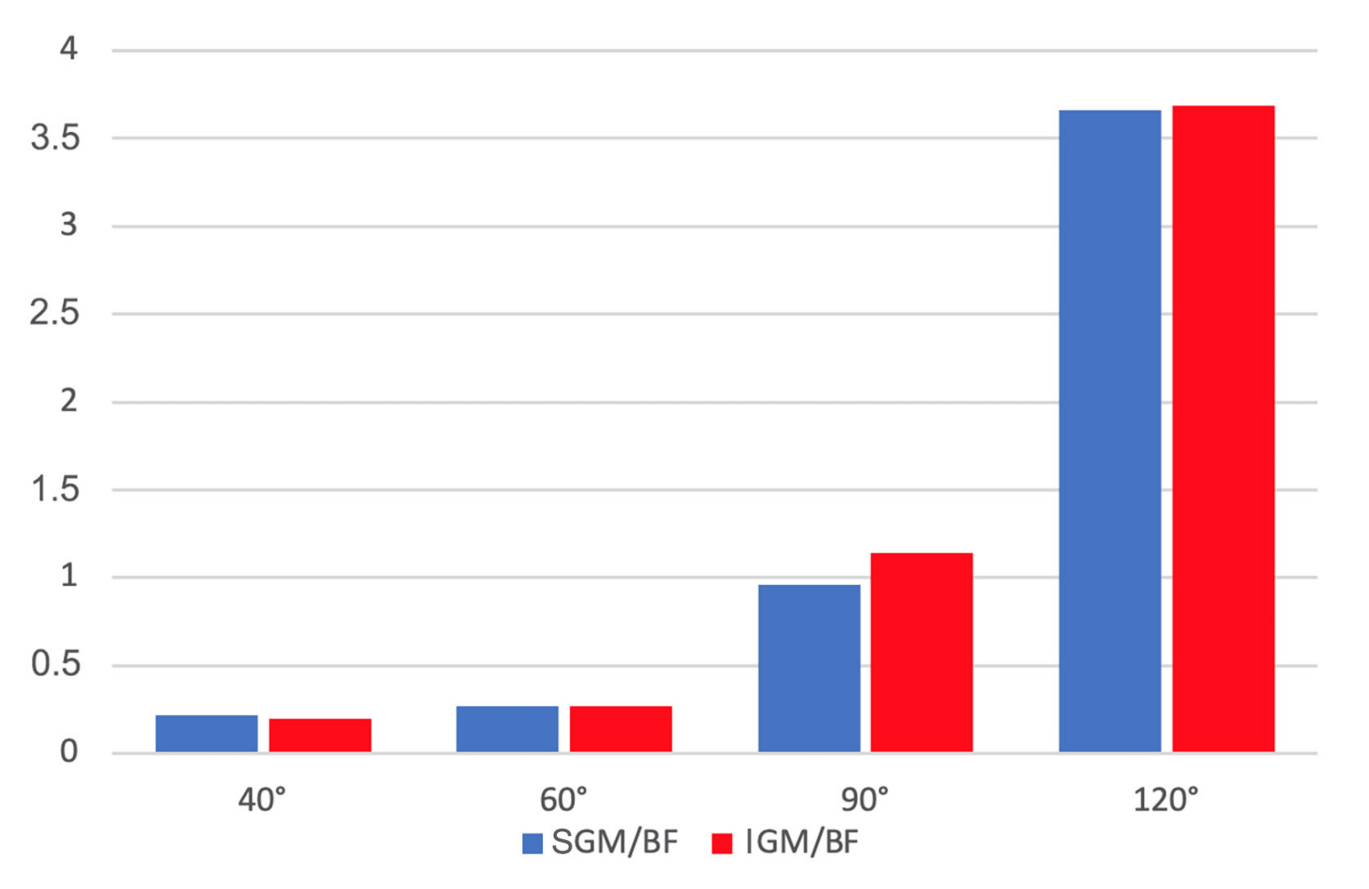
Superior gluteus maximus/biceps femoris (SGM/BF) and inferior gluteus maximus/biceps femoris (IGM/BF) ratios The graph shows how the SGM/BF and lGM/BF ratios (y-axis) change with varying knee flexion angles (x-axis). The data used to construct the histogram were taken from the work of Ho et al. [18]. Image credit: Author Saverio Colonna

In another study [18], the relationship between the superior GM/BF (SGM/BF) and inferior GM/BF (IGM/BF) was investigated during the SBE at four different knee flexion angles. Regarding the SGM/BF ratio, it was found that as the knee flexion angle increased (120° > 90° > 60° > 40°), the ratio also increased, as shown in Figure 5.

Since one theory about exercise-associated muscle cramps is that they occur due to muscle overload and neuromuscular fatigue [21], performing the SBE with reduced Ham activity, achieved by utilizing a high degree of knee flexion, could reduce the incidence of cramps in the Ham muscles doing this exercise.

Hip Variable

To maintain the bridge position at the hip level, an extension torque is always required, with the sole exception being the combination of the lowest TPd values (feet positioned as close as possible to the pelvis), the highest TT values (hip in full extension), and a voluntary effort of knee extension. In this case, a hip flexion torque appears to be required. The torque developed at the hip during the SBE is related to an increasing function of TPd and a decreasing function of TT [12].

Spine Variable

**Figure 6:**
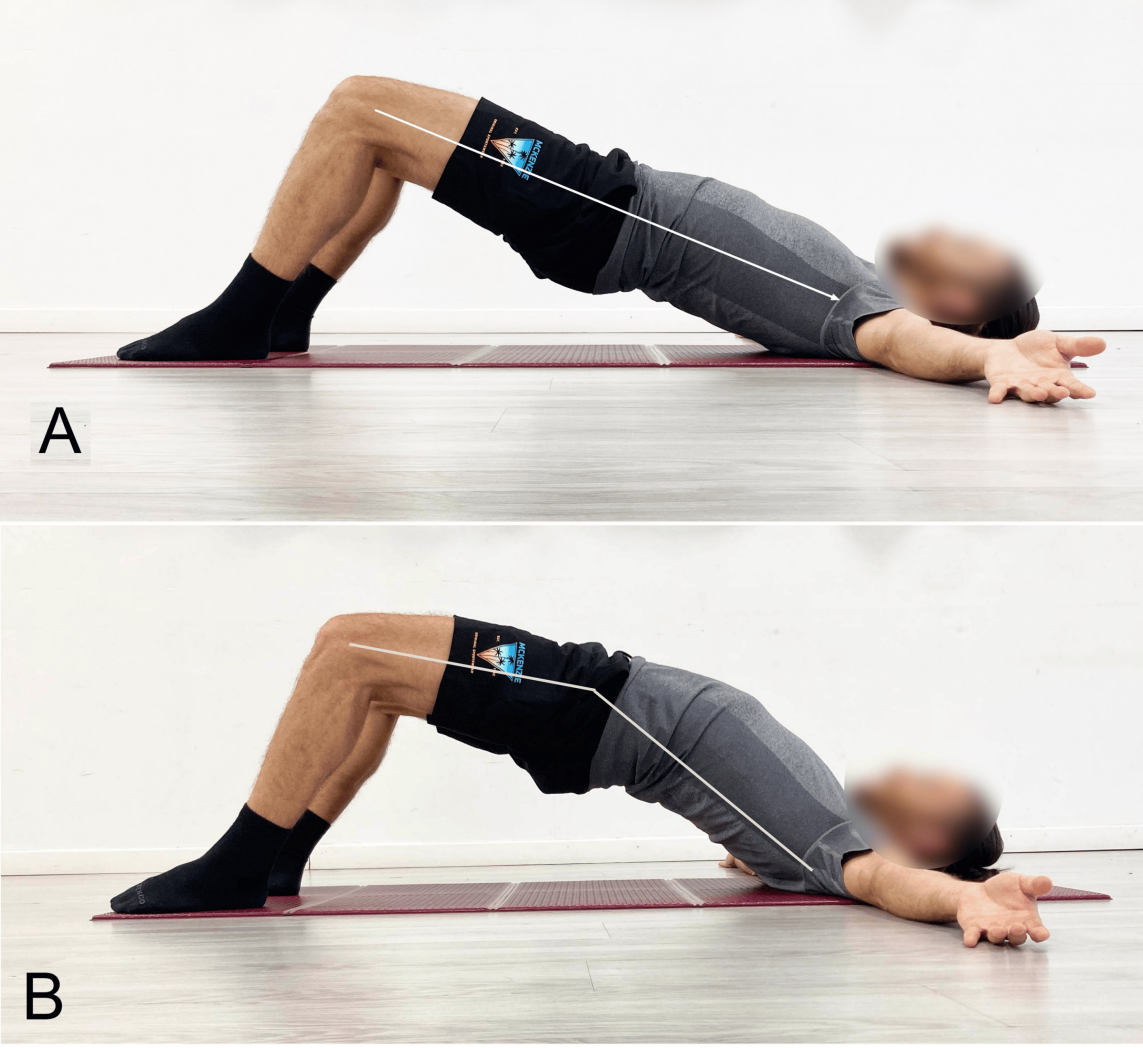
Supine bridging exercise performed with different spinal angles The upper limb was abducted only to better illustrate the difference in the pelvis-spine axis between the two modes of performing the end position of exercise: A) neutral and B) hyperextension. Image credit: Author Saverio Colonna

The positioning of the spine during the execution of the SBE is a topic of debate. Most authors [8,12,22] suggest performing the exercise with the lumbar spine in a neutral alignment (Figure 6A), often without providing a logical justification. For some, the reason is to avoid lumbar hyperextension (Figure 6B) [23].

**Figure 7:**
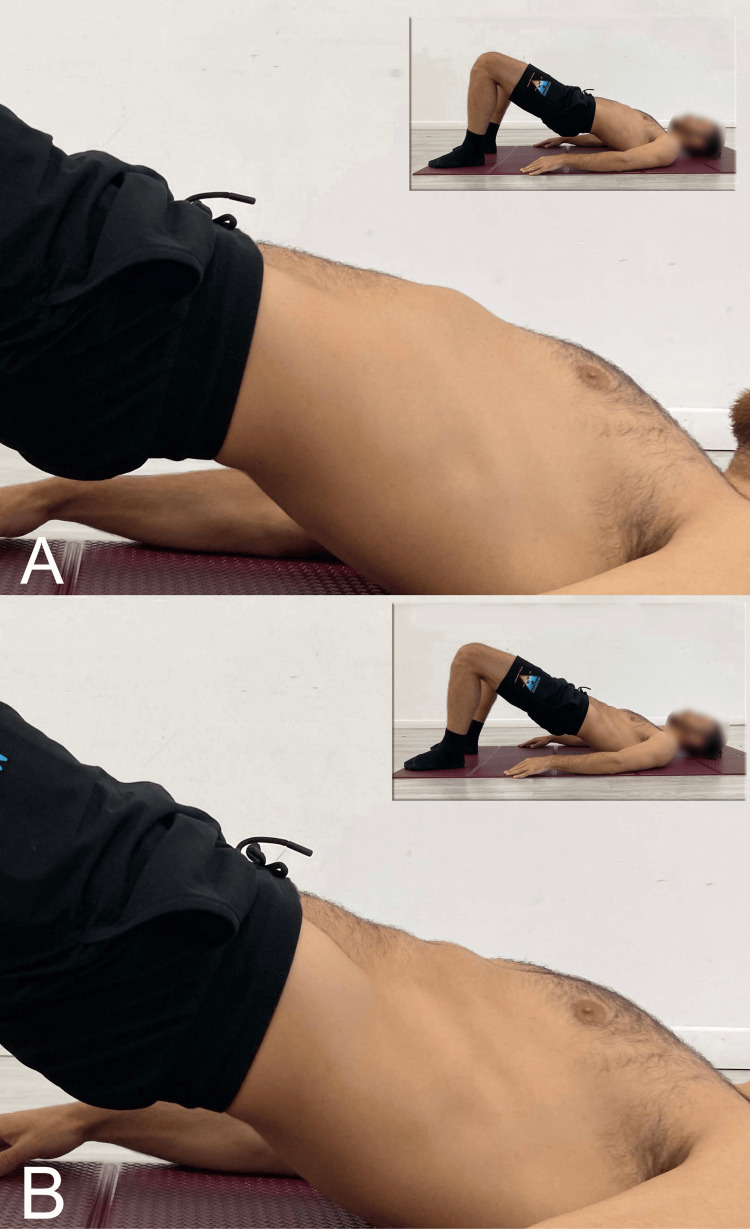
Execution of supine bridging exercise A) Traditional; B) With the abdominal drawing-in maneuver. Image credit: Author Saverio Colonna

In patients performing bridging exercises, excessive and uncontrolled lumbar lordosis and anterior pelvic tilt (APT) are frequently observed due to the dominant hyperactivity of the ES [23]. The repetitive motion associated with this activity could increase compression stress on the lumbar and pelvic regions [24]. Additionally, repeating the bridging exercise without correcting any undesirable lumbopelvic movement may lead to secondary dysfunction [25]. Therefore, some studies have investigated methods to control unwanted lumbar and pelvic movement during bridging. To prevent excessive ES contraction, some authors [26] propose performing bridging with an abdominal activation maneuver called the abdominal drawing-in maneuver (Figure 7).

Others [27] recommend maintaining a straight alignment of the shoulders, hips, and thighs during bridging to prevent excessive APT caused by dominant ES activity. Furthermore, by incorporating isometric hip abduction using an elastic band (thera-band) during bridging, Choi et al. [28] observed increased GM muscle activity along with a simultaneous reduction in APT.

Previous studies conducted on healthy individuals [29] have shown higher %MVIC values in dorsal muscles, such as the ES and Ham, during SBE, and lower %MVIC values in ventral muscles, such as the rectus abdominis (RA) and EO. Conversely, during prone bridging exercises, the %MVIC values of ventral muscles were higher than those of the posterior muscles [29]. The SBE primarily activates the posterior trunk muscles, involving both deep muscles, such as the MF, and superficial muscles, such as the ES [30].

A study [31] on 25 asymptomatic participants investigated the effects of voluntary control of APT, neutral pelvic tilt (NPT), and posterior pelvic tilt (PPT) using visual biofeedback on the EMG of the GM, MF, and Ham during bridging exercises. The muscle activity of the GM and MF significantly differed among the different pelvic tilt controls (APT vs. NPT vs. PPT). GM muscle activity during exercises involving PPT was significantly higher than during exercises involving APT and NPT. Conversely, MF muscle activity during exercises involving PPT was significantly lower than during exercises involving APT. Additionally, the EMG ratios of GM/MF (right), GM/MF (left), and GM/Ham during bridging with PPT were significantly higher than those with APT and NPT. The authors concluded that SBE involving PPT can be recommended as an exercise to selectively enhance GM muscle activity and the GM/Ham and GM/MF muscle activity ratios [31].

Variants of classic SBE

After addressing some methods of executing the classic SBE, the following sections outline variants documented in the literature.

Single-Leg Variant

**Figure 8:**
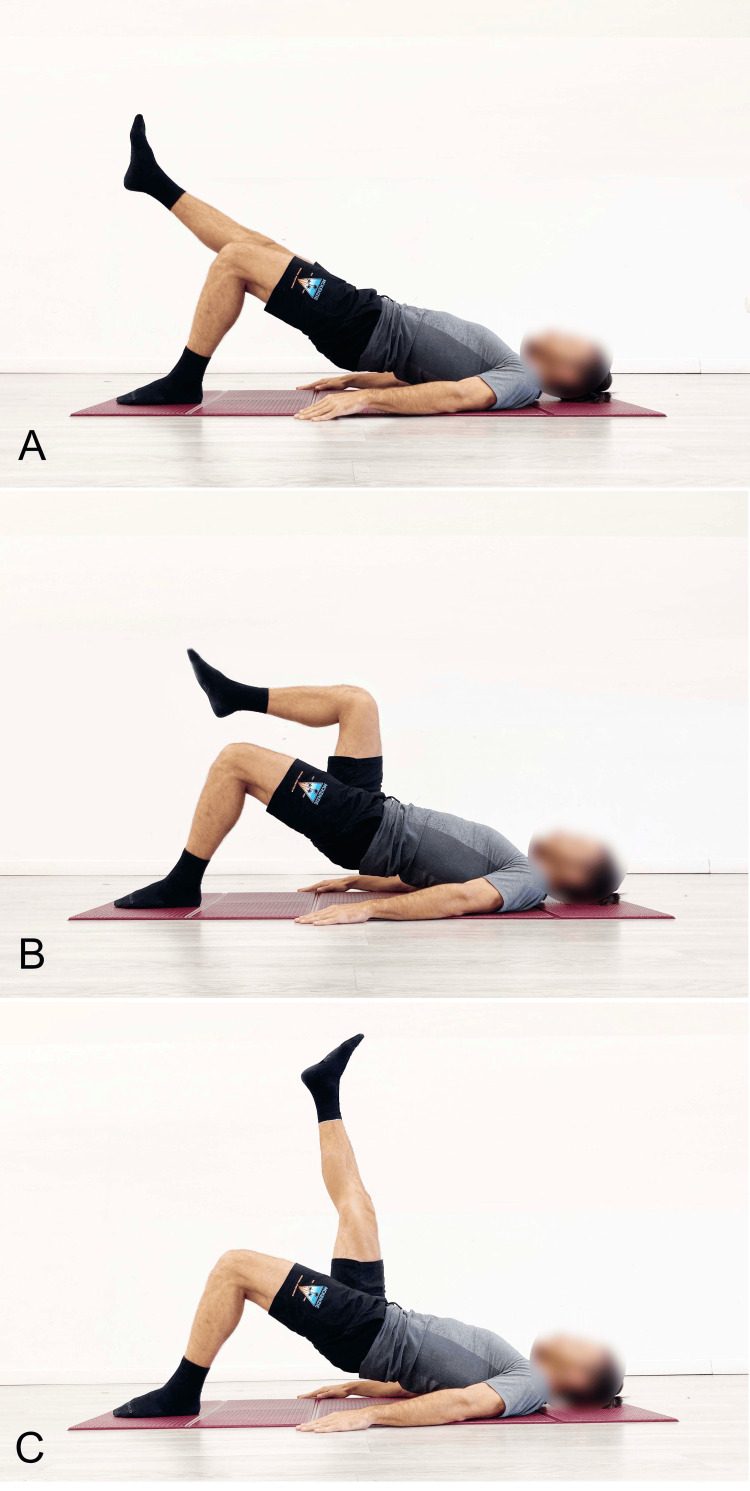
Examples of single-leg stance balance bridge exercises (SLB) A) The limb suspended in line with the trunk; B) Hip and knee flexion; C) Hip flexion with the knee extended. Image credit: Author Saverio Colonna

In the literature, several variants have been proposed to enhance the effects of bridging exercises, including the single-leg support variant, referred to as single-leg bridging (SLB) [32-35]. In the execution of this exercise as proposed in the literature [32], the basic assumption is that the rotation of the pelvis and trunk is maintained in a neutral horizontal plane, meaning that no twisting occurs. The SLB can vary not only in terms of knee flexion angle and DF but also in the position of the suspended leg, which can be aligned with the trunk (Figure 8A), flexed at the hip and knee at 90° (Figure 8B), or vertically aligned (Figure 8C).

**Figure 9:**
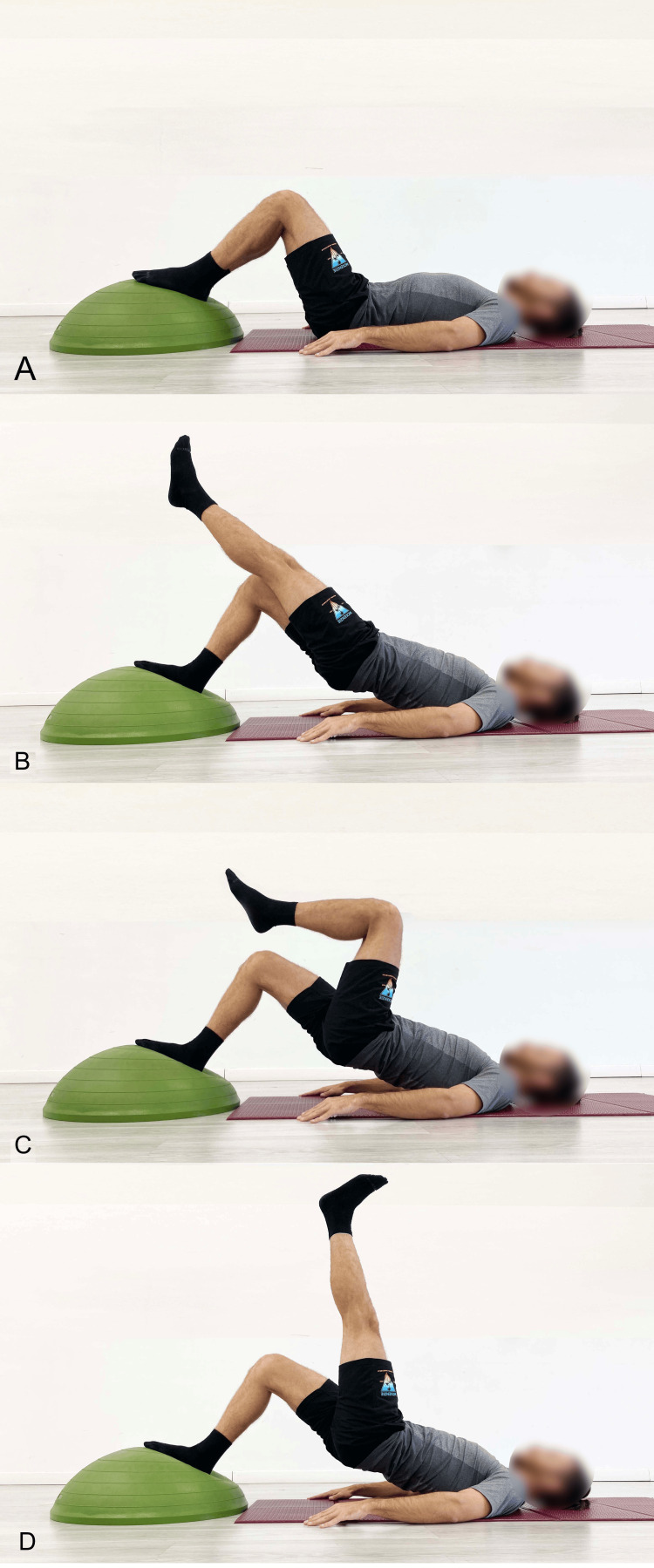
Single-leg bridging on unstable surfaces like a BOSU (both sides utilized) ball A) Starting position with both feet on the surface; B) End position with the suspended leg flexed at the hip and the knee extended, aligned with the spine; C) End position with the suspended leg flexed at both the hip and knee; D) End position with the suspended leg flexed at the hip and the knee extended, perpendicular to the ground. Image credit: Author Saverio Colonna

The described variations in the suspended leg position logically result in increased activation of the quadriceps and hip flexors in the version with an extended knee, with consequent stretching of the Ham. García-Vaquerov et al. [32] reported that when bridging exercises were performed with single-leg support, the rotational torque in the trunk increased, requiring greater activation of the trunk muscles to maintain a neutral lumbar position. Recent research has studied the effects of bi-leg bridging and SLB exercises with foot support on an unstable surface, such as a BOSU (both sides utilized) ball (Figure 9), to add an additional dynamic stabilization challenge [33,34].

The results indicate that transverse abdominal activation, evaluated via ultrasound, was not significantly different between single- and double-leg bridging or between stable and unstable surfaces [34].

However, in the study by Stevens et al. [33] using EMG, it was reported that, regarding abdominal muscles, during all forms of SBE-classic, Swiss ball, and single-leg-relative RA activity was significantly lower than relative oblique muscle activity. During SLB, contralateral EO activity was also significantly higher than contralateral internal oblique (IO) activity, while ipsilateral EO activity was significantly lower than ipsilateral IO activity.

SLB produced the second-highest activation of gluteal muscles among nine rehabilitation exercises examined and the highest activation of Ham muscles (40% MVIC) [5]. A surface EMG of hip muscles in the traditional SLB position with 90° knee flexion demonstrated 40% activation for the GM and 47% for the Gmed [5].

Compared to double-leg bridging, SLB showed higher activation of the Gmed, GM, and Ham; however, MF muscle activation did not differ significantly [36]. Slightly higher results were reported in a subsequent study [16], showing 51.01% MVIC for the GM and 57.81% MVIC for the Gmed, while BF activity showed a notable increase to 75.3%, significantly higher than the 40% MVIC reported by Ekstrom et al. [5]. Based on these findings, SLB can be considered an effective exercise for strengthening the GM and Gmed without external overload, providing a safe and convenient means of improving hip joint stability.

Regarding anterior trunk muscle activation, a study [35] investigated potential differences in muscle activation during traditional SBE and modifications such as single-leg support on a stable surface and single-leg support on an unstable surface (BOSU ball) [35]. The results showed greater bilateral IO activation during SLB compared to double-leg bridging, with the contralateral IO being significantly more activated than the ipsilateral IO. Adding an instability element, such as the BOSU ball, increased IO activation compared to the neutral condition but not significantly compared to SLB. The rectus femoris (RF) was more activated during SLB, both with and without an unstable surface, compared to double-leg bridging; however, there was no significant difference between SLB on stable versus unstable surfaces [35]. Additionally, during SLB with left foot support, the right RF was more active, though this did not reach statistical significance [35]. In the same study, evaluations of posterior vertebral muscles revealed greater right-side ES activation during both double-leg bridging and SLB with left foot support. However, the differences between types of bridging were not statistically significant [35].

A common clinical observation during SLB is Ham cramps, possibly due to a combination of gluteal muscle weakness and high Ham activation. Clinically, Ham cramps often prevent sufficient repetitions of the exercise, potentially reducing its contribution to gluteal strengthening. An EMG study on gluteal exercises reported Ham cramps in multiple subjects during SLB on both stable and unstable surfaces [20]. Some authors [18] suggest that to reduce the occurrence of cramps during SBE, the exercise should be performed with the feet positioned very close to the glutes, increasing knee extension torque at the expense of flexion torque.

SBE With Hip Adduction

Among the various bridging variants found in the literature, there is one that incorporates isometric hip abduction and adduction against resistance.

Park et al. [37] demonstrated that integrating limb movement during bridging exercises enhances the activity of the IO and MF muscles compared to traditional SBE, with a greater effect observed during bilateral hip movement compared to unilateral movement. Hip abduction and adduction movements contribute to the transmission of force to the lumbar muscles via the anterior superior iliac spine portion of the pelvis [4].

**Figure 10:**
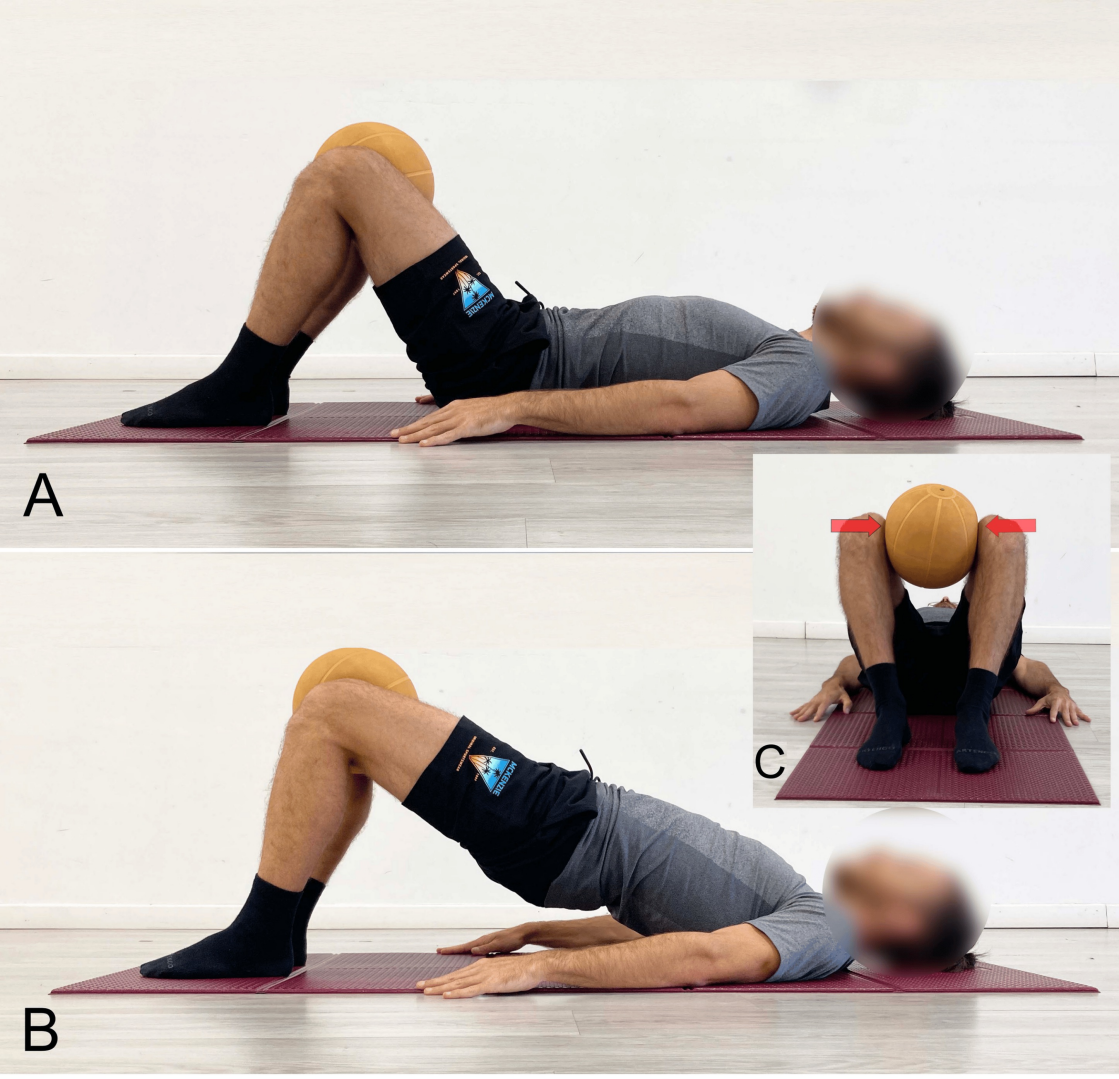
Bridging with the addition of adductor muscle activation using a ball A) Starting position; B) End position, lateral view; C) End position, foot-level view. Image credit: Author Saverio Colonna

A study [15] electromyographically examined, in healthy subjects, the differences between traditional bridging, bridging performed with simultaneous activation of the adductor muscles against elastic resistance (a ball placed at the knee level, see Figure 10), and bridging performed with simultaneous activation of the abductor muscles against elastic resistance (theraband), also placed at the knee level. The results indicate a significantly greater activation (although the study does not provide statistical significance) of the adductor muscles during bridging with adduction compared to traditional bridging. Difficult to explain, however, is the response of the Gmed and tensor fasciae latae (TFL), which showed comparable activation levels during bridging with adduction and during bridging with abduction against elastic resistance.

Even more puzzling is the behavior of the GM, which showed higher activation during bridging with adduction compared to bridging with abduction [15]. This response is not consistent with the physiological principle of reciprocal inhibition. Reciprocal inhibition is the automatic inhibition of the antagonist alpha motor neuron elicited by the contraction of the agonist muscle. This phenomenon is ubiquitous and fundamental in humans as it plays a critical role in the control of voluntary movements [38].

According to Lee [39], when comparing electromyographically the traditional SBE with SBE combined with simultaneous activation of the adductor muscles (ball between the knees) and SBE combined with simultaneous activation of the abductor muscles (elastic band between the knees), it was found that the bridging exercise with adductor activation increases the activation of the TrA compared to the traditional SBE. Additionally, there was a significant negative correlation between the adductor magnus and the Gmed, as well as between the adductor magnus and the RF, during the traditional bridging exercise. A significant positive correlation was observed between the adductor magnus and Gmed and the TrA during bridging with simultaneous adductor activation. There was also a significant positive correlation between the adductor magnus and the Gmed, RF, and between the Gmed and the RF during bridging with abductor activation.

The authors [15] concluded that when performing bridging exercises for TrA activation, incorporating hip adductor contractions is more effective than traditional bridging or bridging with added hip abductor muscle contractions. During bridging with the knee extended and supported by a sling, simultaneous activation of hip adduction combined with hip extension resulted in greater activation of the RA, TrA, and ES compared to abductor muscle activation combined with hip extensors [40].

SBE With Hip Abduction

**Figure 11:**
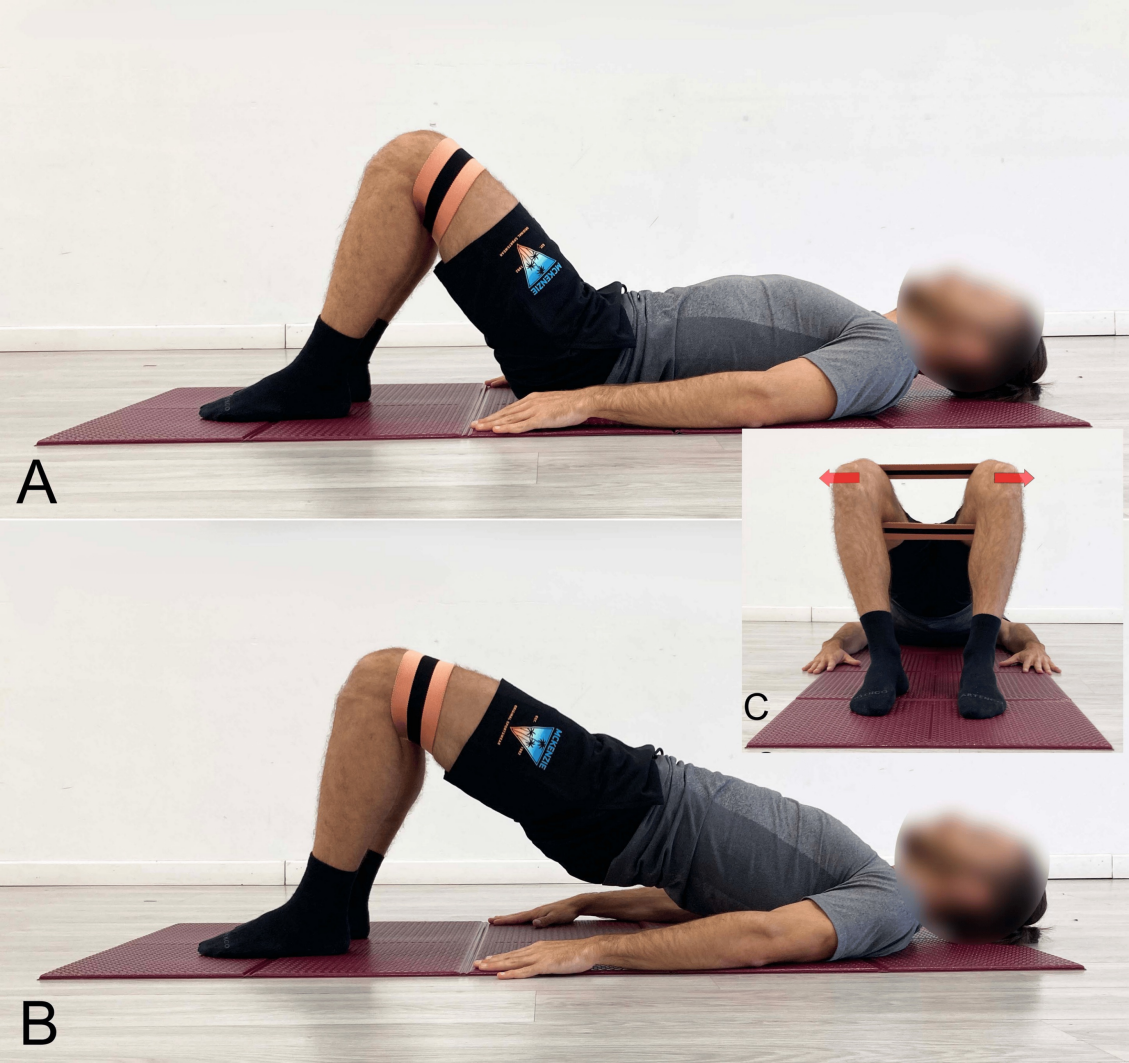
Bridging exercise with added activation of the abductor muscles using an elastic band A) Starting position; B) End position, lateral view; C) End position, foot-level view. Image credit: Author Saverio Colonna

In sports performance, particularly in sprinters, the importance of hip extensors and abductors for performance enhancement and the reduction of ligament injuries at the knee during landing is well-documented [41,42]. In the SBE variation, the integration of an additional resistance plane, such as hip abduction against the resistance of an elastic band positioned at the knees (Figure 11), limited the total load that could be lifted by hip elevation compared to the traditional execution of the exercise [43].

The objective of one study [23] was to investigate, in healthy subjects, how the activity of the ES and GM muscles, as well as the angle of APT, changed when different angles (0°, 15°, 30°) of hip abduction were used during the SBE. Results indicated that bridging with 30° hip abduction could be recommended as an effective method to selectively facilitate GM muscle activity, minimize compensatory ES activity, and reduce the angle of APT.

Another study [28] investigated the effects of performing bridging with concurrent isometric contraction of the hip abductors using elastic resistance (theraband) positioned at the knee level, on pelvic tilt angle and relative muscle activity of the GM, Ham, and ES in healthy subjects. Performing bridging with simultaneous isometric abduction resulted in greater GM activation, while Ham and ES activation did not significantly change. In terms of GM/Ham and GM/ES ratios, no significant differences were observed, but the addition of isometric abduction reduced pelvic anterior tilt by approximately 20%.

A further study [15] evaluated the effects of incorporating simultaneous activation of the hip abductor muscles against elastic resistance at the knees compared to traditional SBE in healthy individuals. The results suggested greater activation (although statistical significance could not be extracted from the study) of the Gmed and TFL, while GM activation showed comparable results between the two trials.

The right hip in 30° abduction during left SLB influenced muscle activation by inducing greater activation of the right IO, RF, and ES, with statistical significance achieved only for ES. The MF showed greater activation on the left side but without statistical significance [35].

To engage these extensor/abductor muscle groups, which are part of the posterior and lateral myofascial chains [44], the literature has proposed both the barbell bridge (hip thrust) and glute bridge with a barbell, incorporating isometric contraction against elastic resistance (theraband) in abduction [43]. EMG results, which showed a large effect size, demonstrated that the addition of resistance abduction significantly increased upper GM (UGM) activity compared to lower GM (LGM) activity in both the barbell bridge and glute bridge modalities [43]. Contrary to expectations and with a large effect size, the glute bridge elicited significantly greater muscle activity than the same exercise with abductor activation against the elastic band [43].

Bilateral SBE With Elevated Upper Spine Support

**Figure 12:**
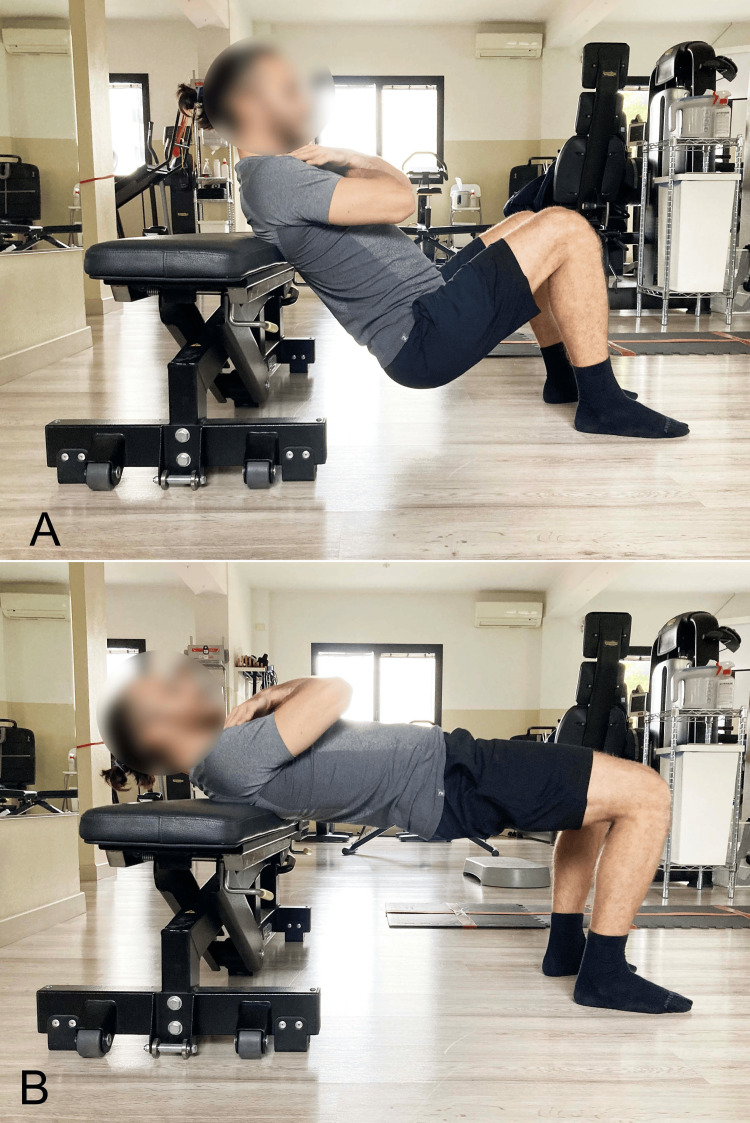
Barbell hip thrust with dorsal support on a bench A) Starting position; B) End position. Image credit: Author Saverio Colonna

**Figure 13:**
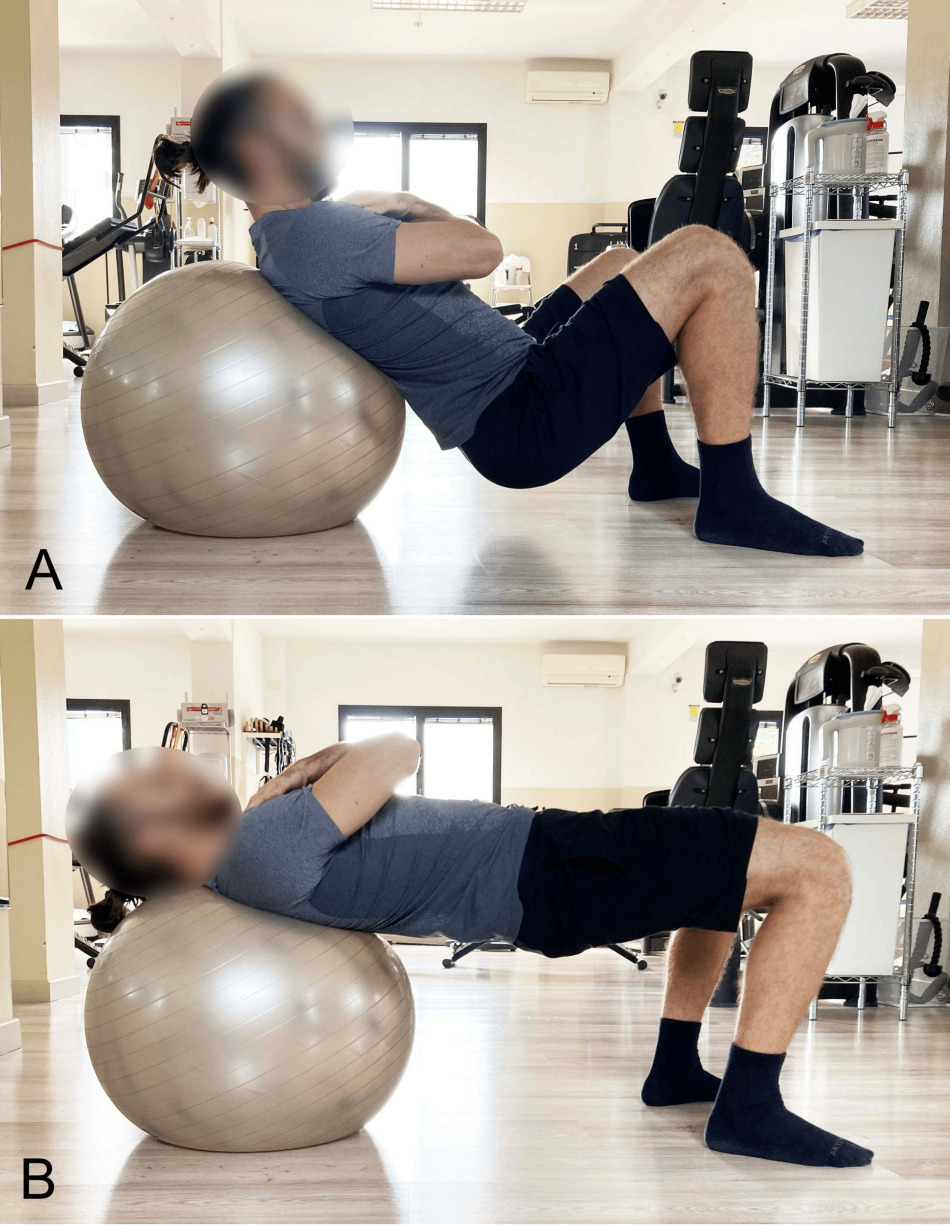
Barbell hip thrust with dorsal support on a Swiss ball A) Starting position; B) End position. Image credit: Author Saverio Colonna

During traditional SBE, the shoulders are typically supported on the floor at the same level as the feet. A variation of this setup is the barbell hip thrust (BHT), where the upper thrunk is supported on an elevated structure above the ground, usually a bench (Figure 12) [45] or a Swiss ball (Figure 13) [46].

**Figure 14:**
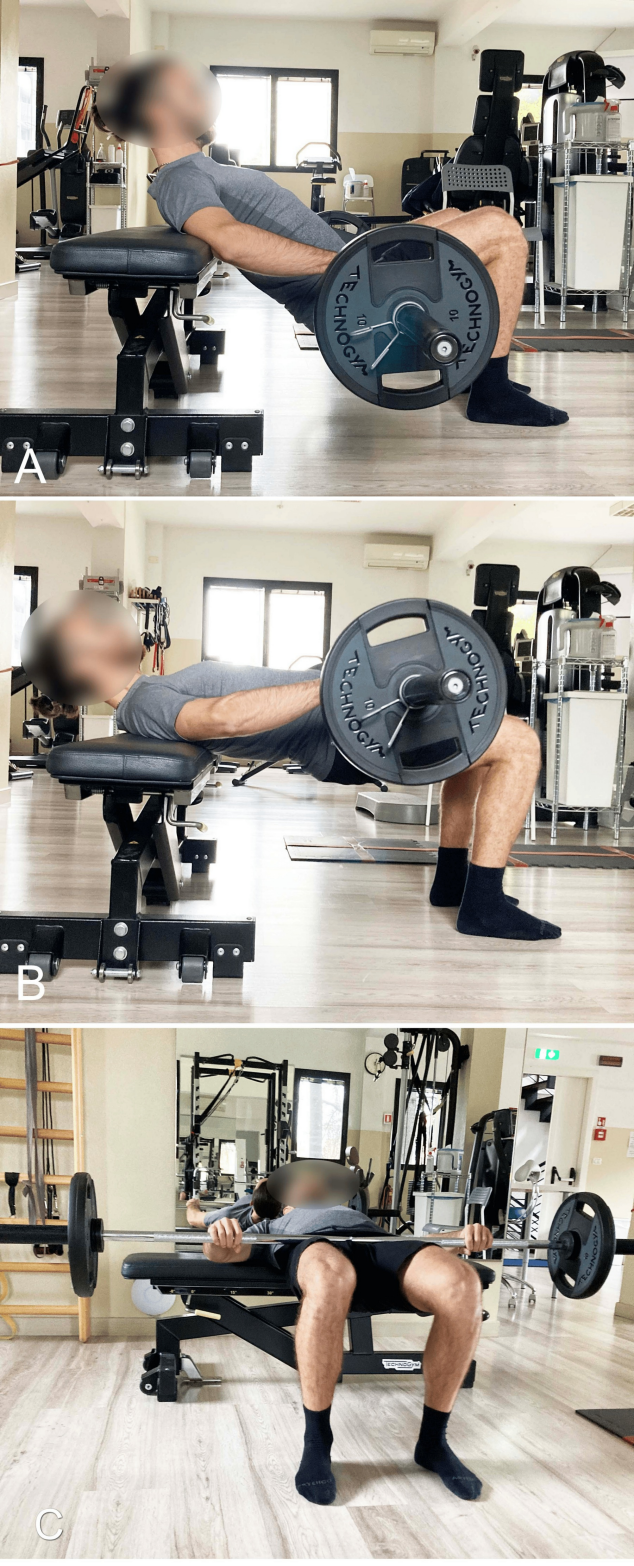
Barbell hip thrust A) Starting position with the barbell; B) End position; C) Foot-level view of the final position. Image credit: Author Saverio Colonna

The BHT, first described in 2011 by Contreras et al. [45], is an exercise that can also be performed with free weights. It involves executing a hip extension with a barbell positioned at the level of the hips while lying supine, with the upper back supported on a bench (Figure 14). In this position, the shoulders are at a height greater than the feet, and the knees are flexed at 90°. The concentric phase of the movement begins with the glutes in contact with the ground and ends when the trunk-pelvis-thigh segment is parallel to the ground, a position achieved with the hips in a neutral position. The exercise can also be performed with the pelvis raised 5-6 cm higher, reaching a state of hip hyperextension through increased glute contraction [45].

The range of motion during maximum physiological hip extension is approximately 20° beyond the neutral position [10]. Moving within this range through active gluteal contraction can maximize GM activation. It has been demonstrated that the EMG of the maximal voluntary isometric contraction of the GM progressively increases as the hip moves from flexion to the extreme degrees of extension [47].

The greatest hip extension torque during the SBE occurs when the hip is nearly fully extended [48]. In this position, the GM is recruited more than at any other angle within the range of motion [10,49,50]. However, it has been shown that an increased hip extension range of motion with weak glutes enhances the anteriorly directed force on the femoral head [51,52]. Therefore, proper exercise progression and attention to ensure gluteal control of the movement are necessary.

In the BHT exercise, unlike the ankle joint, which is minimally involved, dominant extension moments have been observed at the knee, hip, and trunk/pelvic joints, with those at the hip being greater than those at the knee and trunk/pelvis. A recent study [53], evaluating the kinematic differences in vertical displacements between BHT and SBE with an overload, defined as barbell glute bridge (BGB), found that BHT is more suitable for sports requiring force application at smaller hip angles or larger ranges of motion. Specifically, BHT demonstrated greater positive and total vertical impulses compared to BGB, suggesting superior properties for sports requiring high force application per unit time, such as weightlifting. BHT is particularly interesting for practitioners seeking high force application near full hip extension because its range of motion is smaller and closer to complete hip extension, where GM activation is very high.

In addition to the kinematic evaluation, the difference in EMG of the UGM, LGM, GMed, BF, and vastus lateralis (VL) muscles was assessed between BHT and BGB [54]. This study hypothesized that BHT would elicit significantly greater activation of the LGM, GMed, BF, and VL compared to BGB; BGB would elicit significantly greater activation of the UGM compared to BHT. Although the results indicated a statistically significant difference between the two exercises, data analysis confirmed only part of the hypothesis [54]. Consistent with the hypothesis and with large effect sizes for both peak and average results, BGB required significantly greater muscle activity of the UGM compared to BHT, while BHT elicited significantly greater muscle activity of the VL compared to BGB.

Contrary to the hypothesis and with large effect sizes, BGB, rather than BHT, elicited significantly greater activity of the LGM for both peak and average results. Additionally, BGB, rather than BHT, required significantly greater activity with a large effect size for the average response of the Gmed. Although not statistically significant but with moderate effect sizes, BGB elicited greater activity for peak results in Gmed and for average results in BF. No statistically significant differences in BF activation were observed between BHT and BGB for peak results. In BGB, overloads can include a barbell positioned at the level of the pelvis (Figure 14) [54] or an elastic band [55].

Another recent study [43] evaluated the BHT exercise with and without the addition of simultaneous activation of abductor muscles performed against an elastic band positioned at knee level. The results indicated significantly greater activation of the UGM with hip abduction against elastic resistance.

The aim of another study [10] was to compare the EMG activity of UGM, LGM, BF, and VL between the back squat and BHT in 13 adult women. Compared to the back squat, BHT elicited significantly greater mean and peak activation of UGM, LGM, and BF. No significant differences were found in mean or peak EMG activity of the VL. The conclusion was that, using an estimated 10 RM (repetition maximum, or RM refers to the maximum number of repetitions a person can perform with a given load before reaching muscle failure) loads, BHT activates the upper and LGM and BF to a greater extent than the back squat.

The effect of a six-week training program involving BHT and front squats in a group of adolescent athletes was evaluated [56]. The results, based on effect size measures, indicate potentially beneficial effects of the front squat for vertical jump height compared to the BHT. No clear benefit was observed for interventions targeting long jump performance. However, potentially beneficial effects were noted for the BHT compared to the front squat in 10- and 20-meter sprint performance.

During bridging exercises with cranial support on a Swiss ball, which elevates the upper body relative to the feet and increases instability, the incorporation of upper limb movements leads to increased trunk stabilization effort by creating internal perturbations and enhancing proprioceptive demands [46]. This study supports the integration of arm movements during bridge exercises as a therapeutic option to preferentially load specific trunk muscle groups. These effects may be more favorably maintained during bridge exercises performed on a Swiss ball [46].

Bilateral SBE With Elevated Foot Support on Stable or Unstable Surface

**Figure 15:**
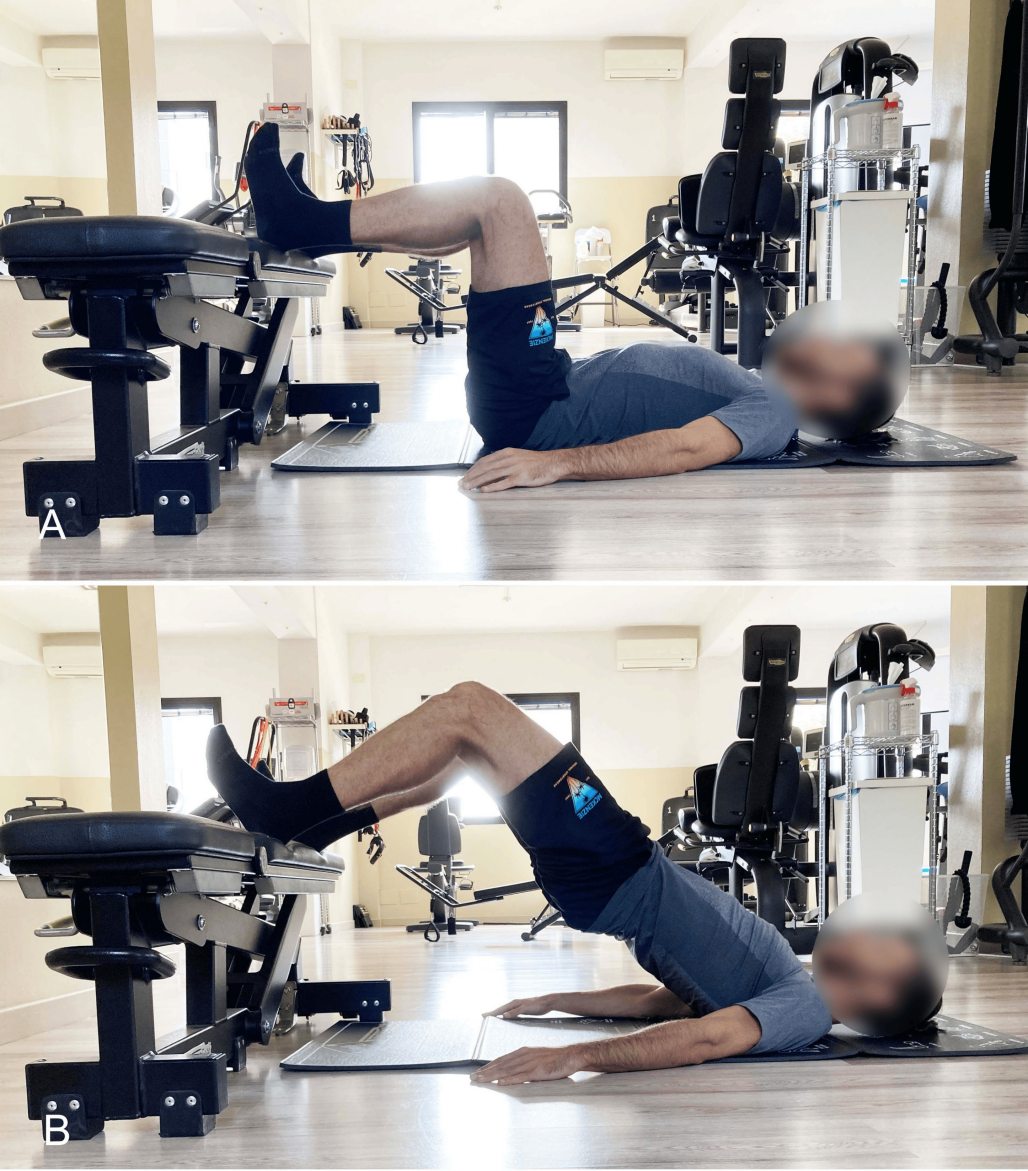
Supine bridging exercise with elevated foot support on a bench A) Starting position; B) End position. Image credit: Author Saverio Colonna

**Figure 16:**
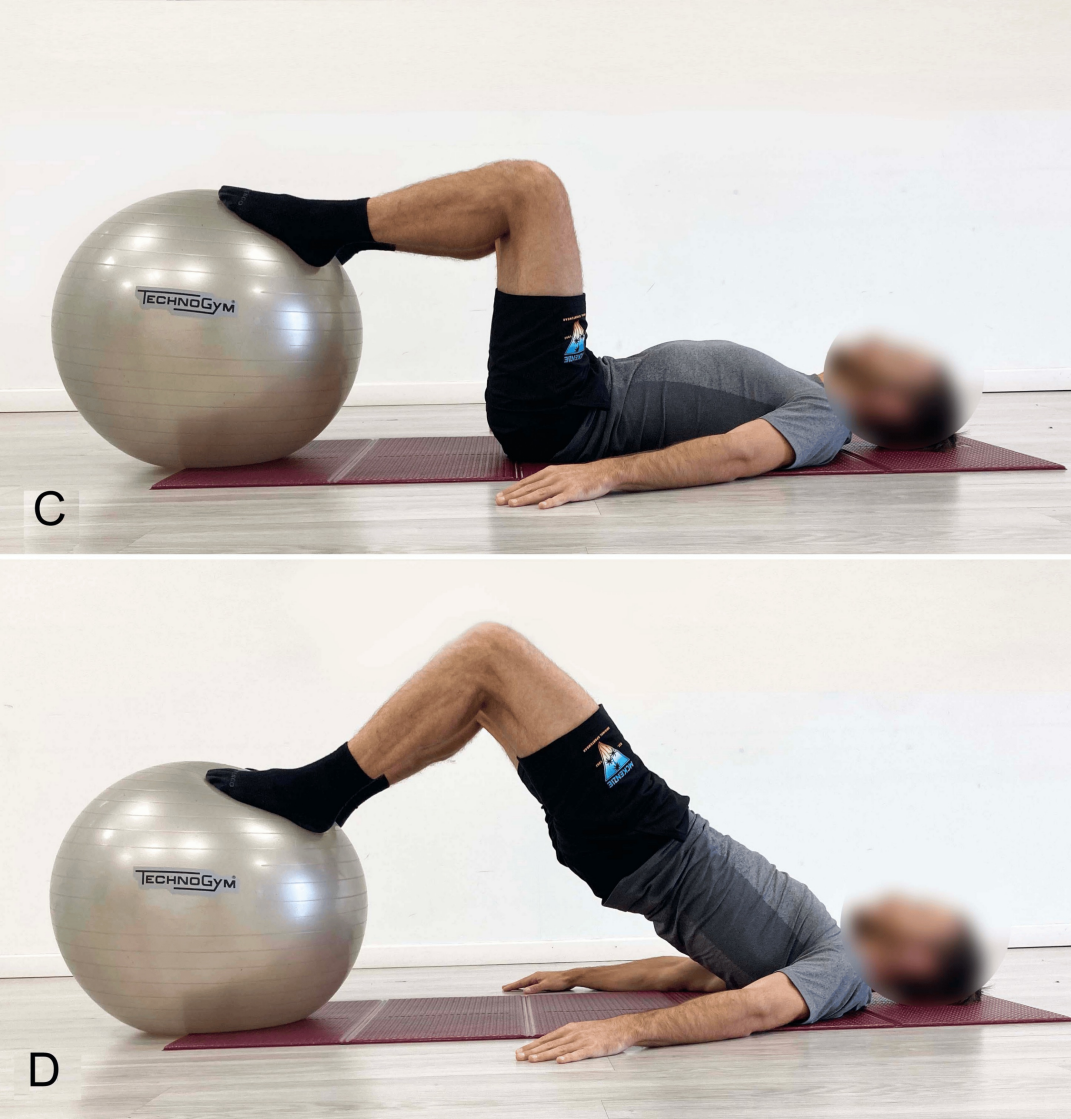
Supine bridging exercise with elevated foot support on the Swiss ball A) Starting position; B) End position. Image credit: Author Saverio Colonna

**Figure 17:**
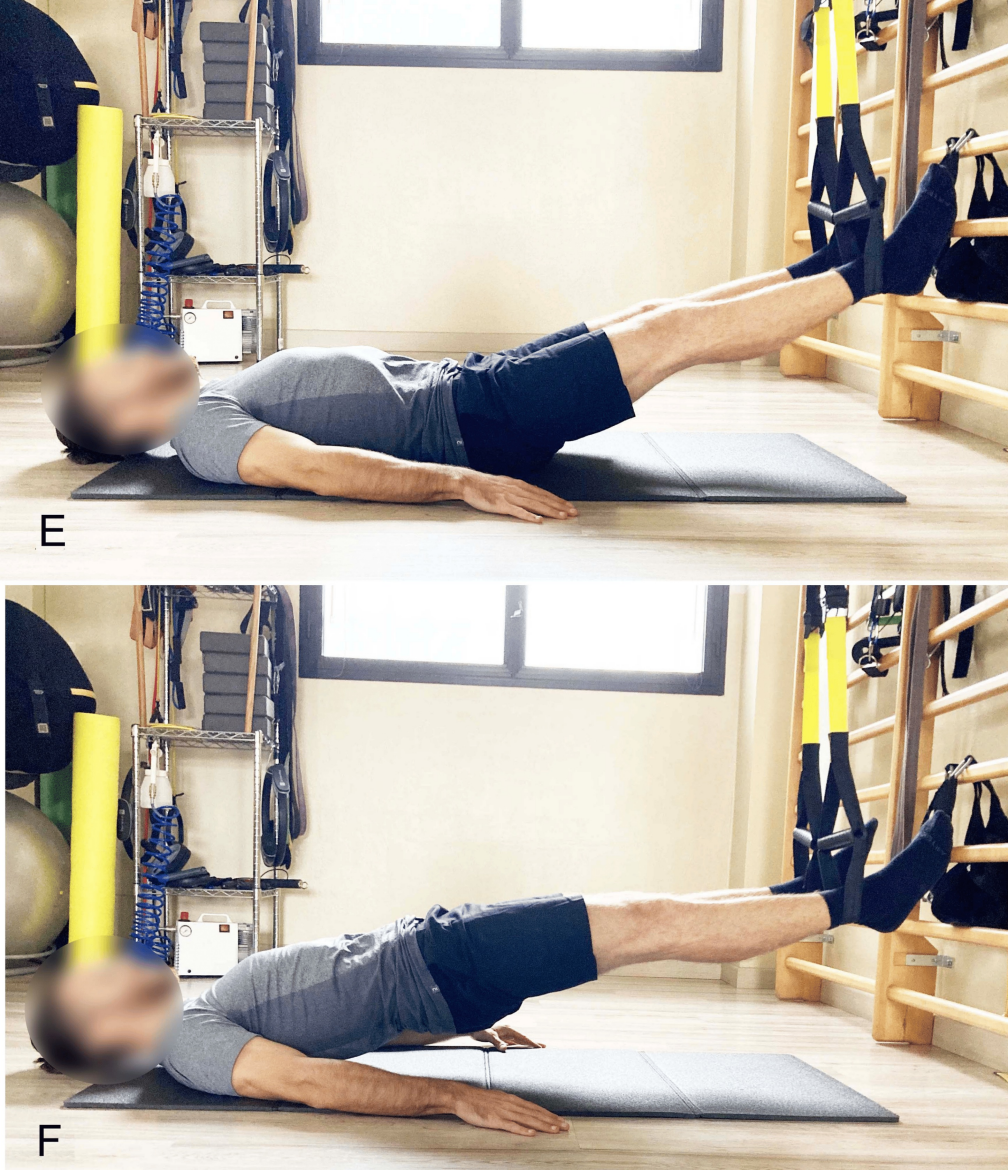
Supine bridging exercise with foot support in slings and extended knees A) Starting position; B) End position. Image credit: Author Saverio Colonna

**Figure 18:**
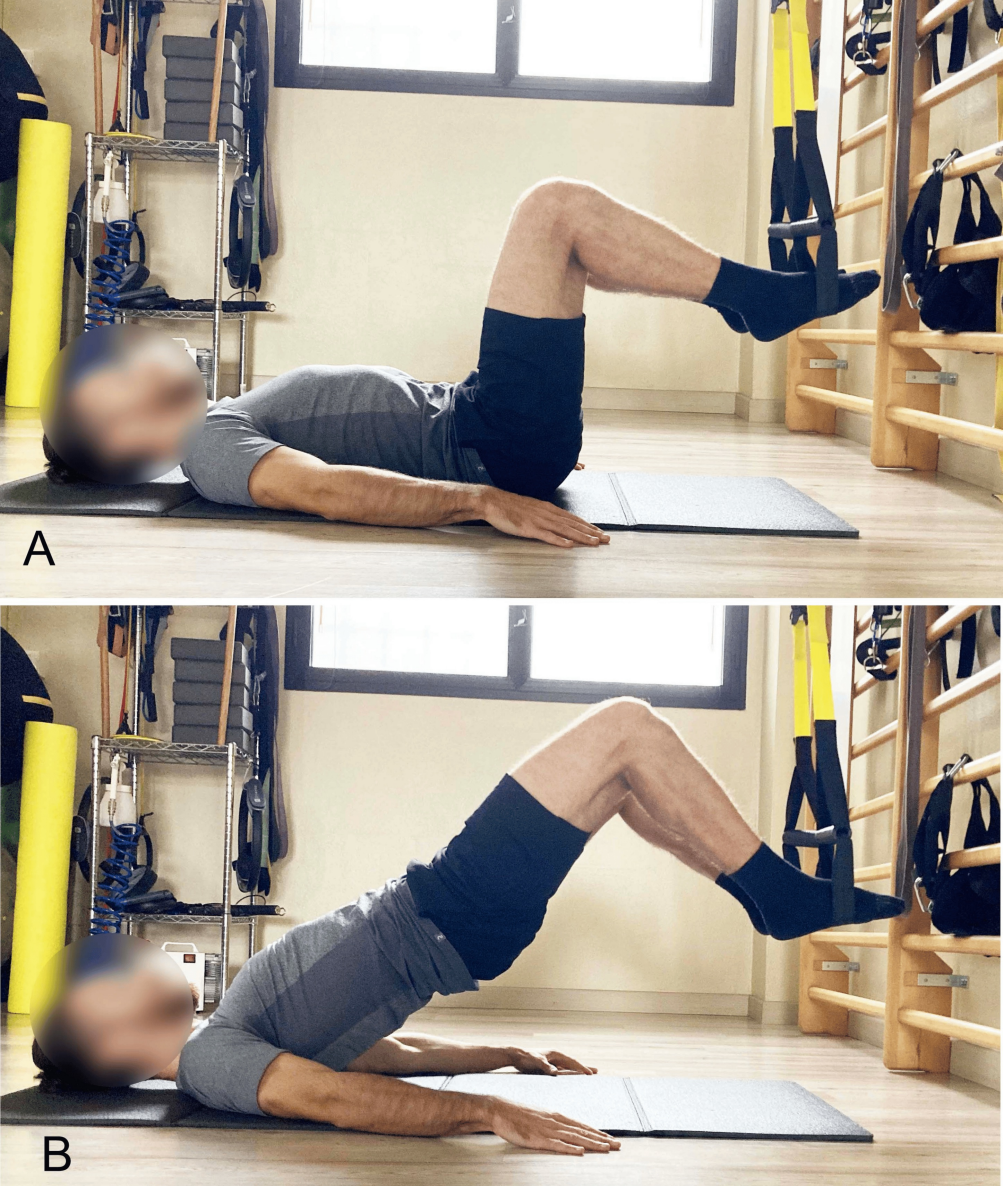
Supine bridging exercise with foot support on slings and flexed knees A) Starting position; B) End position. Image credit: Author Saverio Colonna

Another way to perform the SBE is with the feet elevated relative to the dorsal support, using a bench (Figure 15), a Swiss ball (BBE) (Figure 16), or in suspension (suspension supine bridge exercise, or SSBE) with a sling, with either extended knees (Figure 17) or flexed knees (Figure 18).

The bridging exercise with elevated foot support, such as on a Swiss ball, can be performed with flexed [36] or extended knees [33]. A muscle activation of approximately 30% for the MF and ES is sufficient to maintain lumbar stability during bridging with heels supported on a Swiss ball with extended knees [57]. During a 70-second exercise, a reduced decline in MF EMG was observed, indicating that although muscular endurance is challenged, it does not imply high fatigability, making it suitable for improving muscular endurance [57]. Due to its moderate muscle demands, this exercise might be recommended for trunk stabilization in patients with LBP [57].

In the SSBE, the height of the support appears to influence muscle activation, with greater height leading to increased activation of the TrA, ES [40]. Studies investigating the differences in trunk muscle activation between SBE and SSBE in asymptomatic subjects [33] and those with LBP [30] have consistently reported greater activation of the IO, RA, MF, and ES during SSBE compared to traditional SBE. The BBE showed higher activation of these muscles compared to traditional SBE. Specifically, the MF displayed higher %MVIC values in the SSBE position than in other positions. These results indicate that sling exercises recruit local muscles more effectively than other bridging methods.

Stuge et al. [58] reported that sling exercises enhance the use of local muscles, while other authors [59] noted that sling exercises, as a closed-chain exercise method, improve balance by engaging both local and global muscles, particularly the MF.

A recent article [8] evaluated muscle activation differences during traditional bridging, foot-supported bridging on a BOSU ball, and a Swiss ball in subjects with and without non-specific chronic LBP. The study found significantly greater ES activity during SBE in both pathological and control groups. It also showed that dorsal global muscle activity (e.g., MF) was significantly greater during bridging with foot support on a Swiss ball in participants with LBP compared to asymptomatic individuals. Conversely, local abdominal muscle activity (e.g., TrA) was significantly lower in pathological participants than in non-pathological ones. Considering that local muscle dysfunction leads to intersegmental spinal instability and that increased global muscle activity compromises shock absorption, exercises using a Swiss ball should be used cautiously in LBP rehabilitation [8]. These findings suggest that a patient may develop greater activation of the Ham and increased muscle recruitment when performing bridging exercises on an unstable support surface compared to a stable one. However, some authors [36] disagree on whether SBE performed on unstable foot support surfaces universally improves the recruitment of the lumbar MF, glutes, and Ham compared to stable surfaces.

Consistent with this view, another study [36] found no significant difference in GM EMG recruitment (%MVIC) between unstable foot support conditions (BOSU and Swiss ball) and stable surface conditions for all three bridging exercises evaluated. This suggests that surface stability is not a critical determinant of GM, GMed, and MF recruitment during the bridging exercises studied. Instead, unstable conditions led to greater Ham activation.

Other studies have examined muscle activity during suspension bridging exercises with the sling supported at the ankle and popliteal region rather than underfoot, as in the previously mentioned studies [34,37]. These exercises also incorporated hip abduction without knee flexion. The results suggest that integrating hip movement into bridging exercises may be more effective in facilitating local trunk muscle activity (e.g., IO and MF) and optimizing global and local trunk muscle activities compared to traditional SBE, with bilateral hip movement providing more benefits than unilateral movement [37].

Both traditional SBE and modified versions with extended knees and distal support at the ankle recruit the TrA, but results indicate that sling-based bridging exercises elicit greater TrA activation [34]. Additionally, LBP patients may achieve greater TrA recruitment during the more complex SSBE activity. Clinically, sling-based therapy can be used to train the neuromuscular system of deep spinal stabilizers in LBP patients [34].

SBE and muscle activation

As previously discussed, there are multiple ways to perform SBE, each presenting unique muscle activation patterns. Below is a narrative review utilizing PubMed and Google Scholar without applying any filter regarding the selection date (time period searched) on how different SBE execution modalities influence the activity of the main muscles involved, focusing on the GM, Gmed, Ham, ES, MF, EO, and TrA.

Gluteus Maximus (GM)

The GM is the largest muscle of the hip, representing 16% of the total cross-sectional area [60]. Hip dysfunction (e.g., weakness and limited range of motion) is a factor associated with lower back pain and various lower limb pathologies. There is currently a moderate relationship between hip dysfunction and lower back pathology [61]. Hossain and Nokes [62] proposed a model of dynamic sacroiliac joint instability caused by improper recruitment of the GM and BF muscles, leading to lower back pain.

A very strong relationship has been identified between hip dysfunction and knee pathology [61,63]. Weakness in hip abduction and external rotation has been associated with patellofemoral pain syndrome. Ireland et al. [64] found that women with patellofemoral pain syndrome exhibited 26% less hip abductor strength and 36% less hip external rotation strength compared to controls. Similar trends have been identified by other authors [65-67].

Powers [63] theorized that hip abductor and external rotator weakness may lead to excessive hip adduction and internal rotation, resulting in increased knee valgus. This position can place excessive stress on the knee’s ligamentous structures.

**Figure 19:**
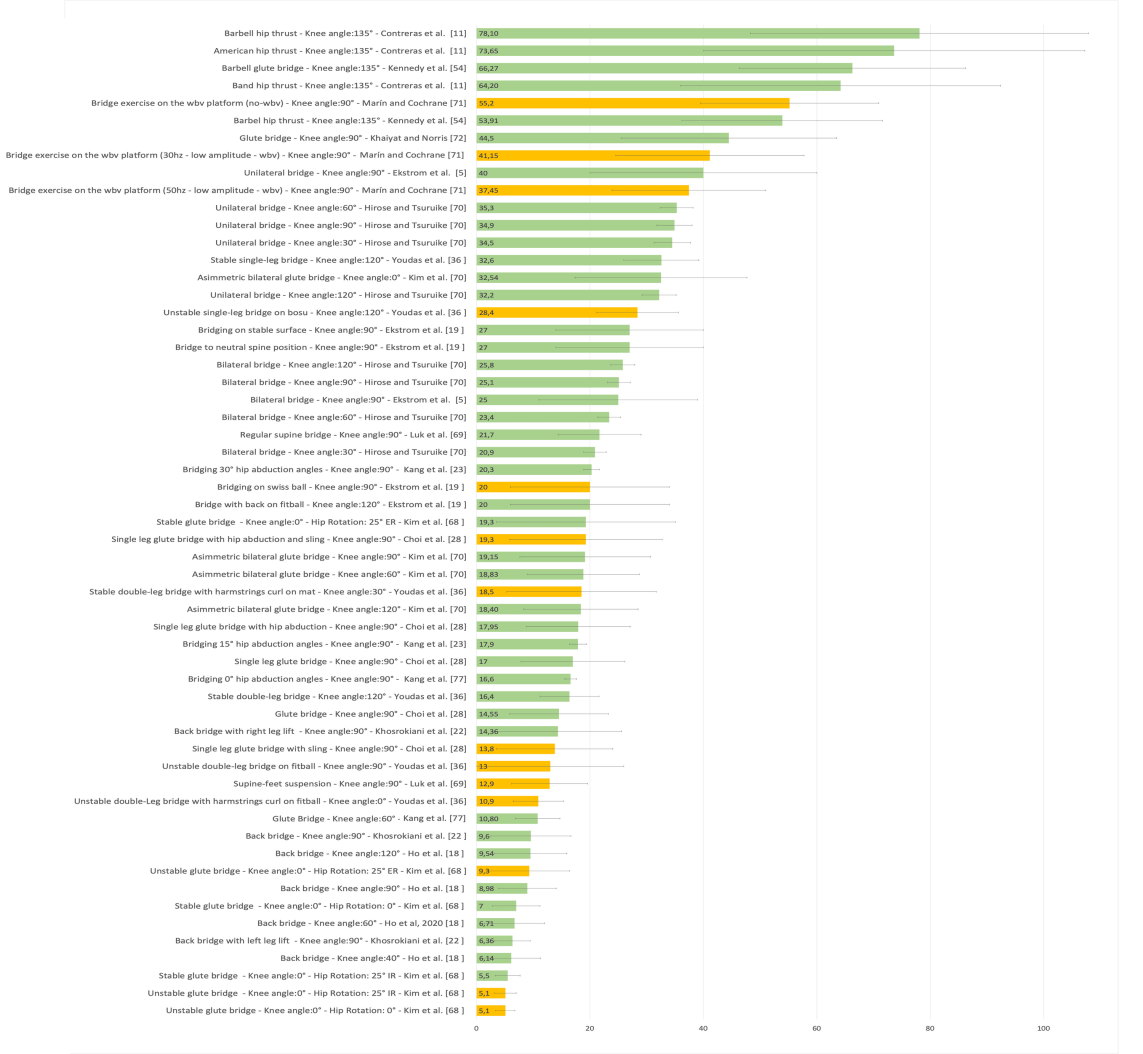
Electromyographic activation of the gluteus maximus during different supine bridge exercise execution modalities proposed in the literature Data are reported as a percentage of maximal voluntary isometric contractions with the corresponding standard deviation (error bars). The green bars represent supine bridge exercise (SBE) performed with cranial or caudal support on a stable surface, while the orange bars indicate that the execution is performed with one of the two support points on an unstable surface. Image credit: Author Saverio Colonna

Figure 19 presents a representative graph of the EMG of the GM, with values expressed as a percentage of MVIC, during different SBE execution modalities proposed in the literature [5,11,18,19,22,23,28,36,54,68-74].

Gluteus Medius (Gmed)

It has been suggested that there is a relationship between a weak or dysfunctional Gmed and lower limb injuries [72,75-77]. The primary injuries associated with a weak or dysfunctional GM are briefly described below.

A person with unilateral or bilateral Gmed weakness may develop a Trendelenburg gait. Normally, the Gmed's role during walking is to keep the pelvis level as one leg swings forward. As one leg moves, the opposite Gmed (on the stance leg) contracts to prevent the pelvis from tilting sideways. In a Trendelenburg gait, the Gmed is unable to maintain the pelvis on the opposite side during single-leg support, causing the pelvis to drop when the swing leg is in the air [78]. This pelvic drop occurs when the Gmed fails to generate enough of an internal hip abduction force to counteract the external hip adduction force that happens during single-leg stance. As a result, individuals with a Trendelenburg gait experience reduced walking efficiency, slower running speeds, and an increased risk of developing lower back pain because the pelvis isn’t stabilized during walking, jumping, landing, or while performing unilateral weight-bearing exercises [79].

In the literature, various pathologies have been linked to deficits in the Gmed. Commonly seen in long-distance runners, Fredericson et al. [80] suggested that iliotibial band syndrome may result from weakness in the Gmed, leading to poor control of thigh abduction and external rotation. According to their hypothesis, this causes increased tension in the iliotibial band, making it more likely to become impinged on the lateral epicondyle of the femur, particularly during the early stance phase of the gait cycle. This impingement is believed to cause the lateral knee pain often associated with iliotibial band syndrome during running. In severe cases, iliotibial band syndrome can persist even when walking or, especially, when descending stairs [80].

Some authors [81] report that a weak Gmed may result from ipsilateral osteoarthritis but can particularly predispose the contralateral hip to developing osteoarthritis. This may be associated with the impaired ability of the Gmed to limit impact during load transfer while walking. Therefore, they recommend strengthening exercises for this muscle [81].

Earl et al. [82] described patellofemoral pain syndrome as an overuse injury characterized by anterior knee pain, which is often exacerbated by activities such as stair climbing, squatting, or prolonged sitting. Dysfunction or inhibition of the Gmed may contribute to reduced hip stability, leading to excessive femoral adduction and/or internal rotation. This results in a larger valgus force at the knee, increasing the lateral forces acting on the patella and contributing to its lateral tracking [82].

Schmitz et al. [83] demonstrated that the Gmed plays a crucial role in maintaining the hip's position in the transverse plane, especially when external rotation forces around the hip are increased. Excessive knee valgus or femoral rotation during landing is a potential mechanism for anterior cruciate ligament (ACL) injuries [84]. Therefore, athletes with strong Gmed control may be better equipped to prevent unwanted adduction and rotational movements during landing. This is particularly important for female athletes, who experience significantly higher rates of ACL injuries (six to eight times greater) and may exhibit more knee valgus and/or hip rotation compared to male athletes [84].

A weakness in the hip abductors, of which the Gmed is a part, may prevent an individual from activating the hip strategy quickly enough to counter a sudden lateral external force. This delay could increase the risk of ankle injuries [85]. Further evidence supporting the role of the Gmed in preventing ankle injuries comes from the same research [86], which found that individuals with hypermobile ankle joints had a delayed activation of the Gmed. This suggests that both a loss of strength and the inability to quickly engage the Gmed may raise the likelihood of ankle injuries.

**Figure 20:**
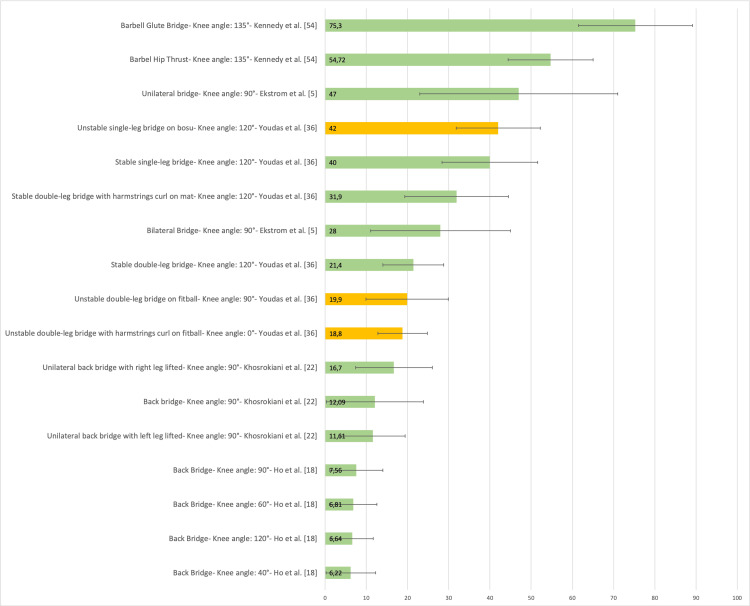
Electromyographic activation of the gluteus medius during different supine bridge exercise execution modalities proposed in the literature Data are reported as a percentage of maximal voluntary isometric contractions with the corresponding standard deviation (error bars). The green bars represent supine bridge exercise performed with cranial or caudal support on a stable surface, while the orange bars indicate that the execution is performed with one of the two support points on an unstable surface. Image credit: Author Saverio Colonna

Figure 20 presents a representative graph of the EMG of the Gmed, with values expressed as a percentage of MVIC, during different SBE execution modalities proposed in the literature [5,18,22,36,54].

Hamstring (Ham)

Ham strain injuries are the leading cause of missed training and playing time in sports that involve running [87]. In professional soccer, for instance, about 20% of players will experience a Ham injury during a season [88], with more than 20% of these injuries recurring [89]. Although the causes of Ham injuries are multifactorial, strengthening the Ham plays a crucial role in injury prevention [90,91] and has been the subject of extensive research [92-95]. The Ham muscles act as agonists to the ACL and serve a protective role when the knee is flexed between 15° and 30°, which is the most common position for ACL injuries [96]. Weakness in the Ham muscles of an ACL-deficient knee can indicate impaired knee function [97].

**Figure 21:**
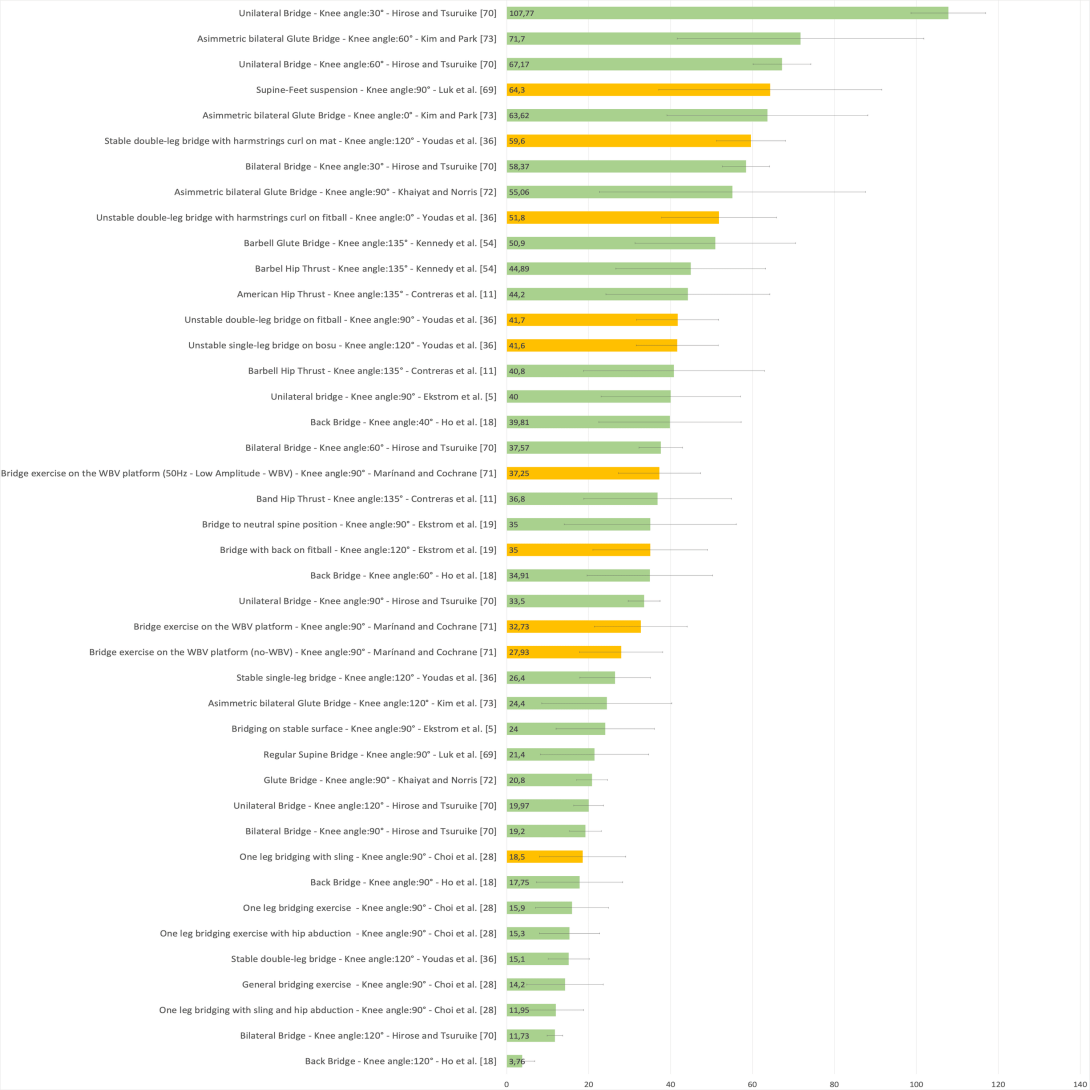
Electromyographic activation of the hamstring during different supine bridge exercise execution modalities proposed in the literature Data are reported as a percentage of maximal voluntary isometric contractions with the corresponding standard deviation (error bars). The green bars represent supine bridge exercise performed with cranial or caudal support on a stable surface, while the orange bars indicate that the execution is performed with one of the two support points on an unstable surface. Image credit: Author Saverio Colonna

Figure 21 presents a representative graph of the EMG of the Ham, with values expressed as a percentage of MVIC, during different SBE execution modalities proposed in the literature [5,11,18,19,28,36,54,69-71,73].

*Erector Spinae* *(ES)*

The ES muscle group plays a key role in maintaining an upright trunk posture. Due to its multiple attachment points, certain parts of the ES have a greater mechanical advantage than others [98]. These anatomical characteristics are clinically significant for individuals with LBP, as the ES muscles are attached to the lumbar vertebrae and directly contribute to the extension of the lumbar spine. Additionally, the thoracic ES (including the thoracic parts of the longissimus thoracis and iliocostalis lumborum) have been shown to cross the lumbar spine and generate forces at the L4-L5 joint [99]. Several studies have found a link between LBP and back muscles that fatigue easily [100-103].

**Figure 22:**
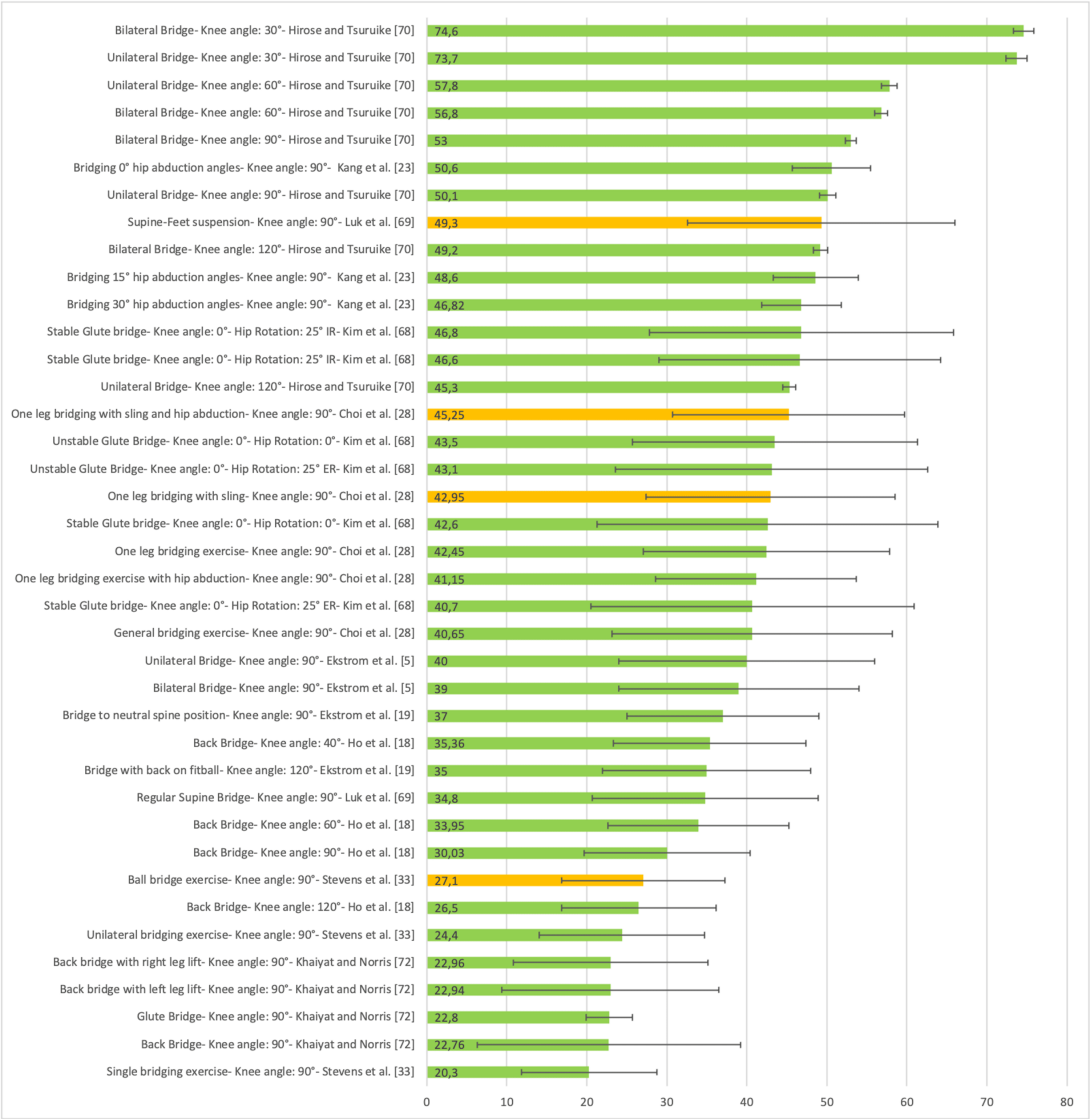
Electromyographic activation of the erector spinae during different supine bridge exercise execution modalities proposed in the literature Data are reported as a percentage of maximal voluntary isometric contractions with the corresponding standard deviation (error bars). The green bars represent supine bridge exercise performed with cranial and caudal support on a stable surface, while the orange bars indicate that the execution is performed with one of the two support points on an unstable surface. Image credit: Author Saverio Colonna

Figure 22 presents a representative graph of the EMG of the ES, with values expressed as a percentage of MVIC, during different SBE execution modalities proposed in the literature [5,18,23,28,33,68-70,72].

Lumbar Multifidus (MF)

The MF is a deeper muscle group with short lever arms that span one or two vertebral segments. It is activated in a length-dependent manner to help stabilize the lumbar spine during everyday activities [98]. Studies have shown that individuals with nonspecific LBP exhibit changes in muscle structure, such as fatty infiltration, in the MF [104-107]. These changes may impair the muscles' ability to generate enough force to stabilize the lumbar spine effectively, especially during movements of the limbs that increase load on the lumbopelvic region.

Evidence supporting this theory includes findings that the MF shows a reduced activation response (50% less activation in the LBP group compared to healthy controls) during a forward-reaching task that increases the flexion moment in the lumbopelvic area [108]. As a result, patients may increase activation of the lumbar extensors as a compensatory mechanism to make up for the weakened role of the MF in providing stability [109,110].

**Figure 23:**
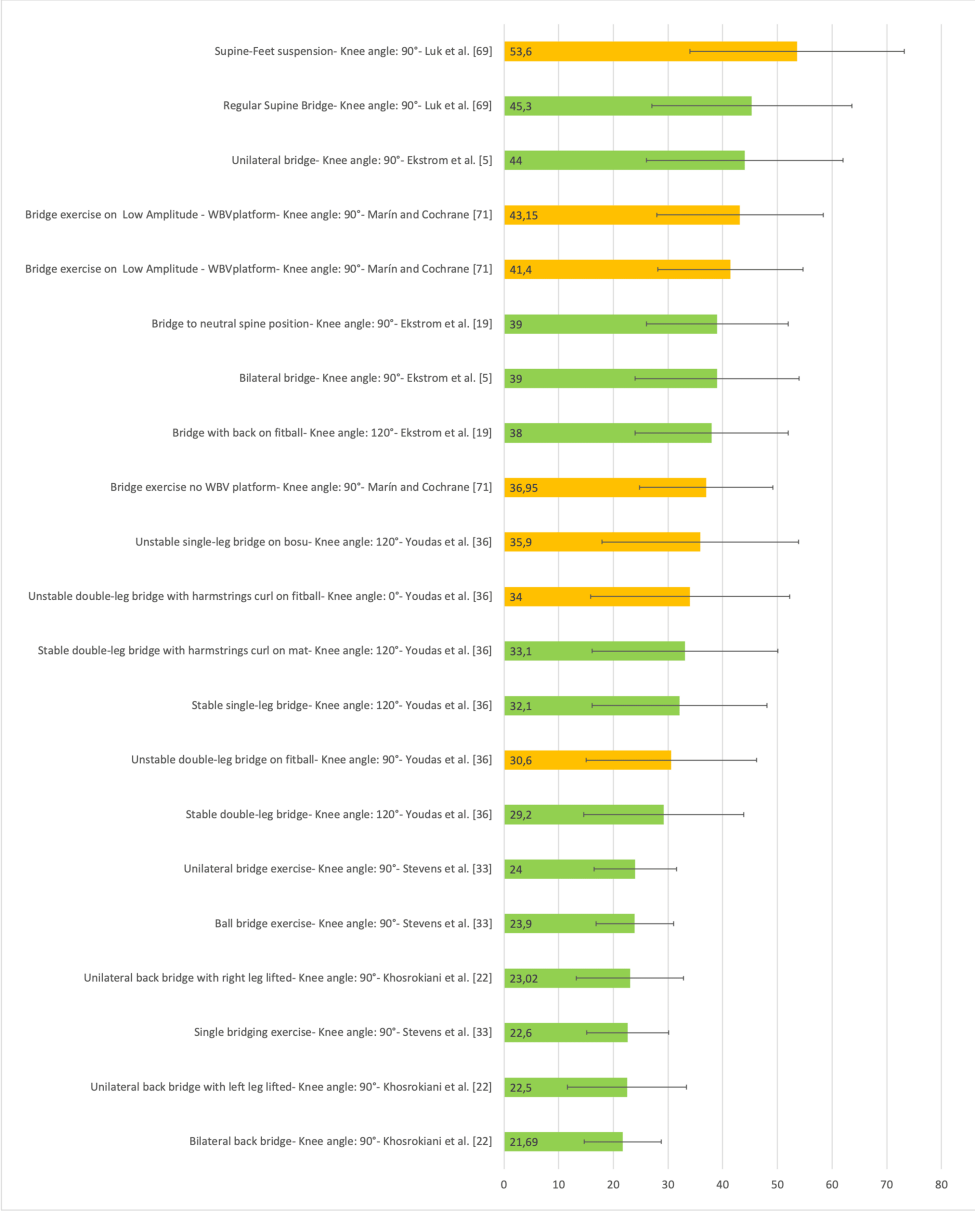
Electromyographic activation of the lumbar multifidus muscles during different supine bridge exercise execution modalities proposed in the literature Data are reported as a percentage of maximum voluntary isometric contraction with the corresponding standard deviation (error bars). The green bars represent supine bridge exercise performed with cranial or caudal support on a stable surface, while the orange bars indicate that the execution is performed with one of the two support points on an unstable surface. Image credit: Author Saverio Colonna

Figure 23 presents a representative graph of the EMG of the MF, with values expressed as a percentage of MVIC, during different SBE execution modalities proposed in the literature [5,19,22,33,36,69,71].

External Oblique (EO)

The abdominal muscles, like EO, are vital in providing core stability for functional movements during most activities [111]. As a key muscle in the core, enhancing the activity level of the EO can improve function and alleviate pain [112]. Research has indicated that individuals with LBP have lower signal magnitudes in the EO muscle compared to those without LBP. Thus, boosting the activity of this muscle can serve as a sign of recovery for the patient [113].

**Figure 24:**
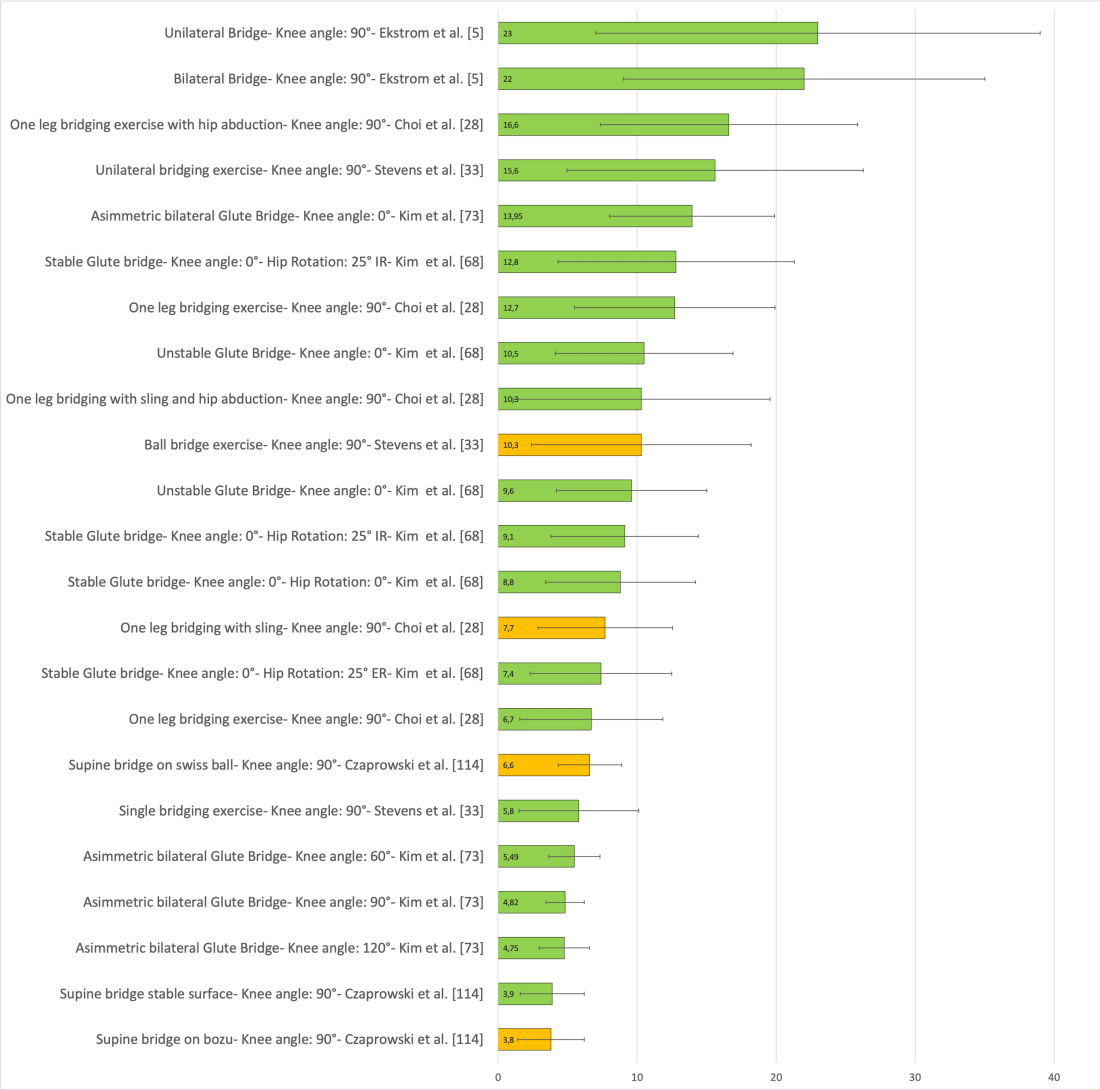
Electromyographic activation of the external oblique during different supine bridge exercise execution modalities proposed in the literature Data are reported as a percentage of maximum voluntary isometric contraction with the corresponding standard deviation (error bars). The green bars represent supine bridge exercise performed with cranial or caudal support on a stable surface, while the orange bars indicate that the execution is performed with one of the two support points on an unstable surface. Image credit: Author Saverio Colonna

Figure 24 presents a representative graph of the EMG of the EO, with values expressed as a percentage of MVIC, during different SBE execution modalities proposed in the literature [5,28,33,68,73,114]. Another study [115] reports EMG data of the EO during the bridging exercise, both bipodal and monopodal, on stable and unstable surfaces. However, the data are not presented in a table because the authors used only the absolute values for statistical analysis rather than percentages of MVIC.

Transversus Abdominis (TrA)

It has been suggested that motor control changes, such as dysfunction in the TrA muscle [116], are linked to a higher long-term risk of LBP. Postural activation of the deepest abdominal muscle, the TrA, is impaired in individuals with chronic recurrent LBP [117] and in healthy subjects experiencing experimentally induced acute LBP [116,118]. These findings suggest that LBP may lead to TrA dysfunction. Conversely, it is also possible that TrA dysfunction could contribute to LBP.

There is substantial evidence indicating that TrA plays a crucial role in providing stiffness between vertebral segments [119] and that its postural activation aligns with this function [118]. Additionally, normal TrA control is impaired in individuals with chronic recurrent LBP, even when they are not experiencing pain at the time [119]. Rehabilitation programs have been designed to target these motor control impairments observed in individuals with LBP, and randomized controlled trials have demonstrated the effectiveness of these approaches [58,120,121].

**Figure 25:**
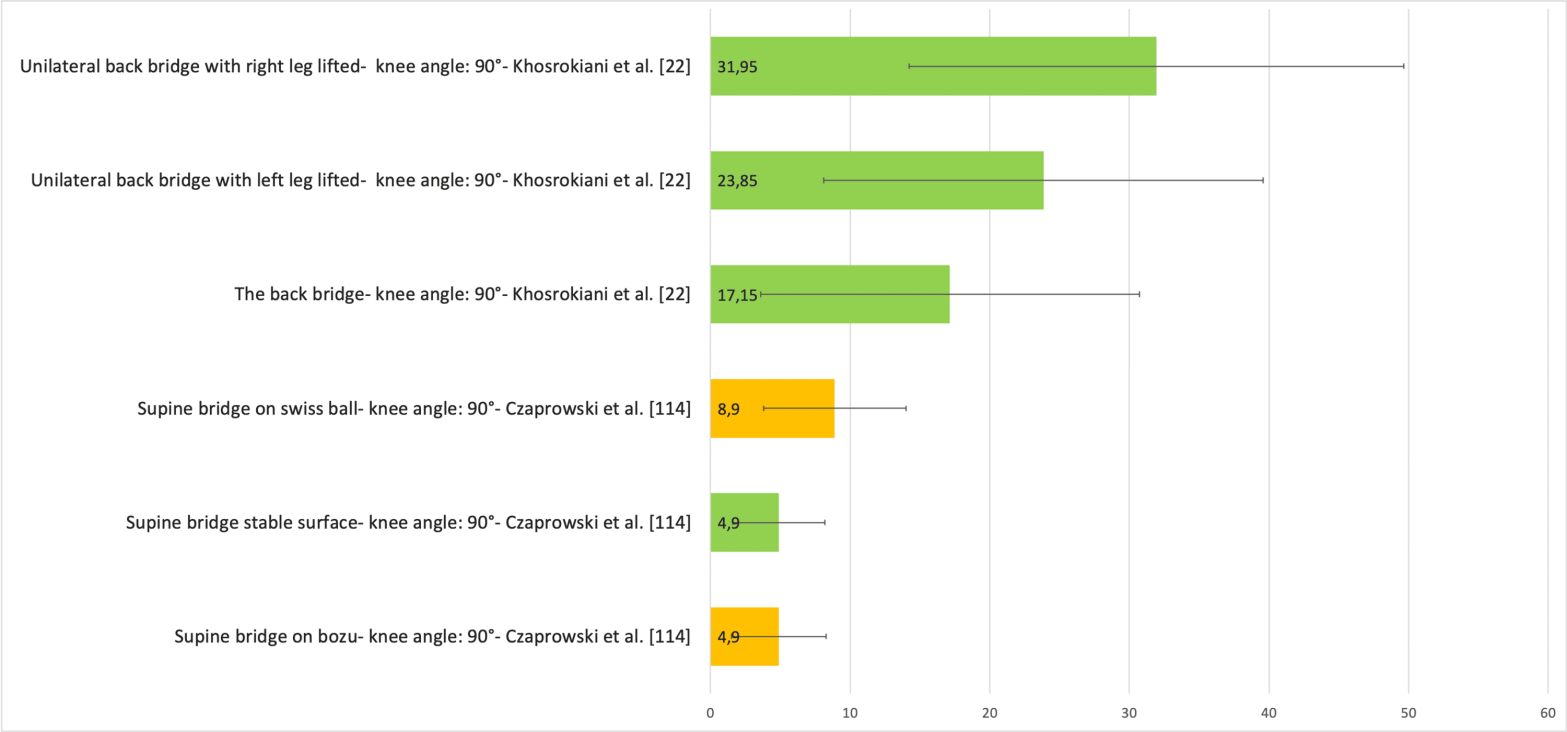
Electromyographic activation of the transversus abdominis muscles during different supine bridge exercise execution modalities proposed in the literature Data are reported as a percentage of maximum voluntary isometric contraction with the corresponding standard deviation (error bars). The green bars represent supine bridge exercise performed with cranial or caudal support on a stable surface, while the orange bars indicate that the execution is performed with one of the two support points on an unstable surface. Image credit: Author Saverio Colonna

Figure 25 presents a representative graph of the EMG of the TrA, with values expressed as a percentage of MVIC, during different SBE execution modalities proposed in the literature [22,114].

## Conclusions

Upon reviewing the literature, a multitude of articles on the subject of SBE can be found. This exercise, widely used in rehabilitation and prevention, offers various execution modalities that selectively engage different muscles. In this review, we aimed to outline, considering most of the modalities present in the literature, the implications of performing the exercise with different angles of the involved body segments, on stable or unstable surfaces, and with cranial or caudal support elevated.

Additionally, to facilitate the selection of the most appropriate SBE execution method for specifically targeting the muscles involved, we have summarized the data in histograms, showing the percentage of EMG relative to maximal contraction in descending order. This article serves as a practical guide or "manual" for utilizing SBE across a variety of rehabilitative contexts, providing insights into how the exercise can be adapted to target specific muscles effectively in different clinical scenarios.
